# Changes in potential distribution and cultivation areas of *Allium victorialis* L. under climate change

**DOI:** 10.3389/fpls.2025.1629527

**Published:** 2025-09-03

**Authors:** Yi Huang, Jian Yang, Guanghua Zhao, Yang Yang, Wuzhi Jiaba, Jialin Li, Tingyong Yang, Jianmin Yao

**Affiliations:** ^1^ Key Laboratory of Biodiversity and Environment on the Qinghai-Tibetan Plateau, Ministry of Education, School of Ecology and Environment, Tibet University, Lhasa, China; ^2^ Sichuan Provincial Forest and Grassland Key Laboratory of Alpine Grassland Conservation and Utilization of Tibetan Plateau, College of Grassland Resources, Southwest Minzu University, Chengdu, China; ^3^ School of Life Sciences, South China Normal University, Guangzhou, China; ^4^ Ecological Security and Protection Key Laboratory of Sichuan Province, Mianyang Normal University, Mianyang, Sichuan, China; ^5^ Grassland Monitoring Center, Ganzi Tibetan Autonomous Prefecture Grassland Work Station, Ganzi, Sichuan, China

**Keywords:** suitability-productivity model, species distribution model, wild vegetables, upper reaches of the Dadu River - Minjiang River, climate change

## Abstract

**Introduction:**

Climate change has profoundly reshaped the spatiotemporal distribution patterns of plants. *A. victorialis*, a wild vegetable with significant edible and medicinal value, is highly favored by residents in the upper reaches of the Dadu River - Minjiang River, leading to its extensive collection and utilization.

**Methods:**

This study simulated the potential distribution of *A. victorialis* in the upper reaches of the Dadu River-Minjiang River using an ensemble model, predicting the impacts of future climate change on its distribution, the migration patterns of the centroid of suitable habitats, and its ecological niche. Additionally, a production dynamic model integrating ecological suitability and nutritional components of *A. victorialis* was constructed to delineate its current and future potential cultivation areas.

**Results and discussion:**

The results demonstrated that the annual temperature range, precipitation in the warmest quarter, and temperature seasonality coefficient are the main factors restricting the potential distribution of *A. victorialis* Multivariate environmental similarity and the most dissimilar variable analysis revealed significant climate anomalies in the study area, indicating that future climate change will have a substantial impact on *A. victorialis.* In the current period, the suitable habitats and high-yield cultivation areas of *A. victorialis* are concentrated in low-altitude river valleys in the upper reaches of the Dadu River - Minjiang River. Under future scenarios, highly suitable habitats and first-class cultivation areas will face shrinkage, with the ecological niche and distribution centroid migrating northeastward. By 2090, under the SSP5-8.5 scenario, the changes will be most drastic: first-class cultivation areas will disappear entirely, and highly suitable habitats will be nearly lost. This study will facilitate the development of suitable management strategies for *A. victorialis* in the upper reaches of the Dadu River-Minjiang River, providing a scientific reference for the sustainable utilization of mountain plant resources under climate change.

## 1 Introduction

Human utilization of plant resources under climate change has long been a focus of interdisciplinary research (Dong, 2018; Huan et al., 2022; Yang et al., 2022a; Horrocks et al., 2024; Anagha and Kuttippurath, 2025; Peng et al., 2025). As a key category of plant resources, edible plants are closely tied to human development (Teixidor-Toneu et al., 2018; Wan et al., 2024). Their growth, distribution, and nutritional composition are shaped by climate, geomorphology, hydrology, and soil, with climate change identified as the primary driver, exerting more profound impacts than short-term non-climatic factors (Avasthi, 2005; Brooks et al., 2006; Harvey et al., 2023; Schlaepfer and Lawler, 2023). Extensive studies confirm that climate change alters edible plants’ habitats, distribution, and nutritional quality (Dong, 2018; Cao et al., 2022; Jiang et al., 2023; Yang et al., 2024a), while extreme climate events further disrupt their metabolites and productivity (Patni et al., 2022).

**Figure 1:**
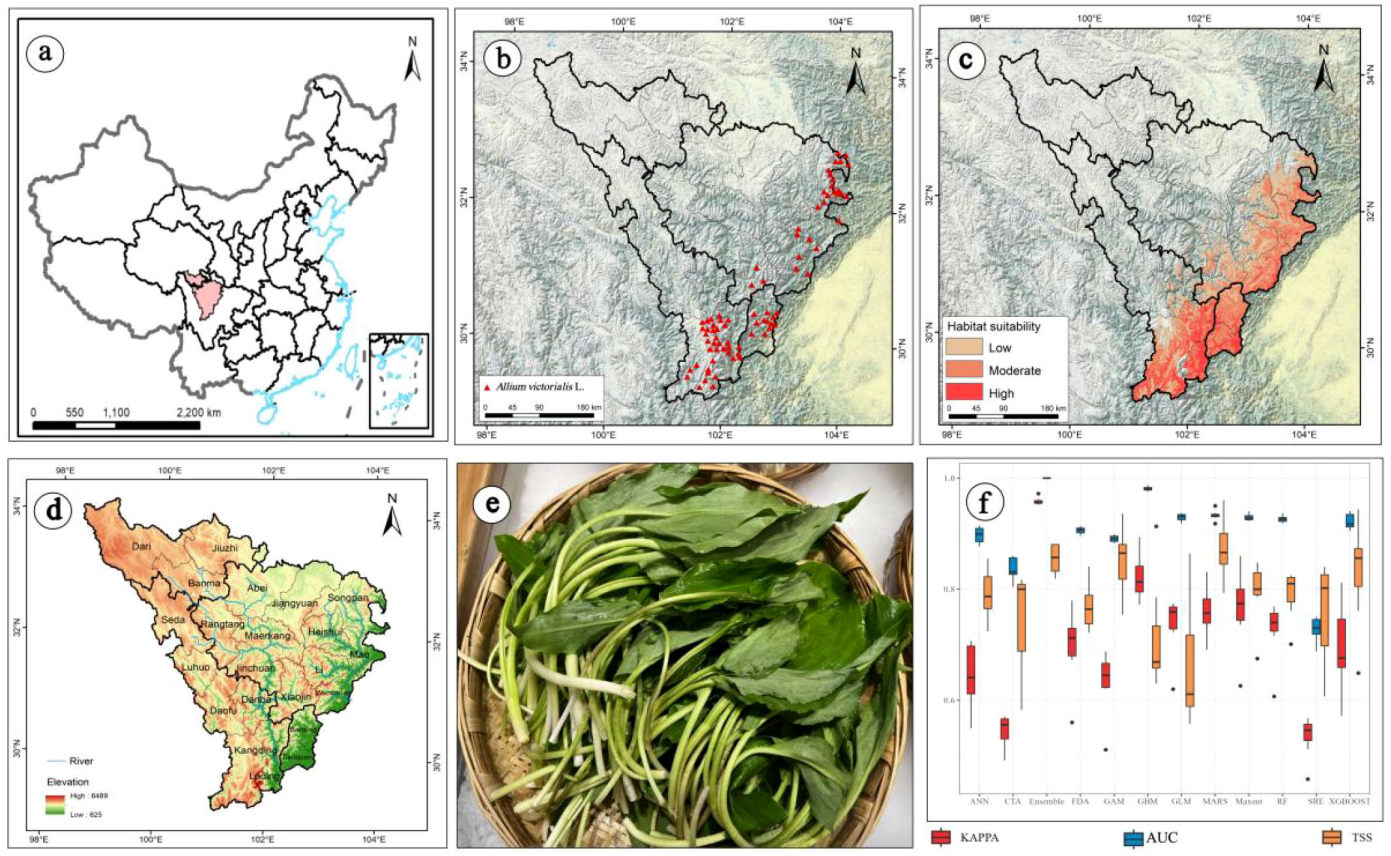
**(a)** Location of the study area in China; **(b)** Distribution records of *A. victorialis* in the upper reaches of the Dadu River - Minjiang River; **(c)** Current potential distribution area of *A*. *victorialis*; **(d)** Overview of the study area; **(e)** Edible parts of *A*. *victorialis*; **(f)** Accuracy evaluation of different distribution models using AUC, TSS, and Kappa statistics. The blue lines in **(d)** represent rivers.

*Allium victorialis* L. (*A. victorialis*), a cold- and shade-tolerant perennial herb in the Liliaceae family, thrives in forests and meadows at elevations of 1200–2500 m across Eurasian temperate regions, with dominant populations in China’s northeastern, northern, and southwestern mountains (Feng, 2023) (**Figure 1e**). In the upper reaches of the Dadu River - Minjiang River regions, it is a culturally and nutritionally vital wild vegetable for Tibetan, Yi, and Qiang communities (Wang et al., 2020), valued for its high iron, pyruvic acid, polysaccharide, and vitamin C content (Kanazawa et al., 1991; Jeong et al., 2005; Lu et al., 2018), as well as diverse amino acids (Yang et al., 2010; Cho et al., 2011). It also has medicinal properties, including hemostatic, analgesic, and liver-protective effects (Cui et al., 2021; Woo et al., 2012; Kim et al., 2012). Although *A. victorialis* has significant dietary and cultural importance, its population numbers are declining in core distribution areas, such as Wenchuan County and Lixian County in Sichuan Province. Consequently, research remains concentrated in the northeastern region, with critical research gaps in the southwestern region (Feng, 2023).

The rapid advancement of species distribution models (SDMs) has profoundly enhanced our understanding of species’ ecological niches and expanded insights into their geographic distributions. Current research on the impacts of climate on species distribution has yielded significant results, applied across fields such as the spatiotemporal distribution and habitats of paleobiota (Huang et al., 2023), potential distributions of energy plants (Cao et al., 2022; Liu et al., 2024b), conservation of endangered species (Huang et al., 2022), and habitats of medicinal plants (Lan et al., 2023; Liu et al., 2024a). The ensemble model constructed by Biomod2 is an R-platform-based multi-algorithm fusion framework. It generates highly comprehensive and less uncertain assessments of species’ suitable habitat distributions by integrating prediction results from multiple single SDMs, thereby improving prediction accuracy and reliability (Thuiller et al., 2009). In recent years, ensemble modeling methods have been applied to predict species’ suitable habitats. For example, Huang et al. (2025a) explored the potential distribution of core and marginal cultivation areas of *Argentina anserina* (a medicinal and edible plant) in alpine regions using an ensemble model and found that the prediction results of the ensemble model were more stable and accurate in the alpine areas. The fruits of *Ginkgo biloba* have high edible value. Feng et al. (2023) explored the habitat suitability of *Ginkgo biloba* through a combined model, and delineated potential suitable production areas by integrating factors such as fruit quality and yield to support the commercial development and utilization of *Ginkgo biloba* fruits. As a comprehensive platform, Biomod2 enhances the accuracy of species distribution predictions by fitting and comparing different models and assessing uncertainties (Bellard et al., 2022).

The upper reaches of the Dadu River - Minjiang River are characterized by a dual identity of “mountain-canyon barriers” and “resource-constrained livelihoods” (Zhang, 2018). The region is marked by high mountains, deep valleys, fragmented terrain, and underdeveloped transportation infrastructure, with most villages relying on winding mountain roads for access (Huang et al., 2025b; Zhang, 2018). Due to topographical constraints and limited connectivity, traditional livelihoods remain central to the daily lives of residents. Arable land is scarce and fragmented into sloped farmland, constrained by steep terrain and the risk of desertification (Hua et al., 2013; Yang et al., 2024b). This area is also a hotspot for global climate change responses (Zhang, 2018), with research indicating that some wild edible plants in this region are highly vulnerable to climatic shifts (Liu et al., 2022, 2025). Existing studies primarily focus on large-scale nutritional assessments or climate change impacts (Li et al., 2020; Guo et al., 2021; Tan et al., 2023), offering limited practical guidance for on-the-ground production practices.

Overall, current domestic research on *A. victorialis* is mainly concentrated in Northeast China, while the high mountain gorge area of Southwest China remains unexplored. Additionally, existing studies on the impact of climate change on *A. victorialis* often focus on a single dimension. Therefore, this study for the first time focuses on the *A. victorialis* resources in the high mountain gorge area of Southwest China. By combining field surveys and model simulations, the following research will be conducted: (1) Predict the current suitable habitat for *A. victorialis* and its changes under different climate scenarios; (2) Analyze the changing trends of the ecological niche and habitat center of gravity of *A. victorialis* under future different climate conditions; (3) Construct a suitability-productivity model to delineate the potential cultivation areas for *A. victorialis*. The results will clarify the ecological requirements of *A. victorialis*, provide a basis for the sustainable management of its resources, and offer a methodological reference for simulating species distribution in mountain ecosystems.

## 2 Materials and methods

### 2.1 Study area

The upper reaches of the Dadu River - Minjiang River are located in the northern part of the Tibetan-Qiang-Yi Corridor, spanning the transition zone from the eastern edge of the Qinghai-Tibet Plateau to the Sichuan Basin (**Figures 1a, d**). Administratively, this region includes the Aba Tibetan and Qiang Autonomous Prefecture, Garze Tibetan Autonomous Prefecture, and parts of Ya’an City in Sichuan Province (Huang et al., 2025b) (**Figures 1a, d**). Dominated by mountain-canyon topography with a significant altitude gradient (1200–5500 m), the landscape slopes from northwest to southeast, transitioning from the Qinghai-Tibet Plateau in the north to the Sichuan Basin in the south. It serves as a critical ecological barrier and water conservation zone in the upper Yangtze River, characterized by a typical plateau-mountain-valley three-dimensional geographic pattern (Zhang et al., 2017; He et al., 2022; Wen et al., 2022).

### 2.2 Collection of species distribution data and environmental variables

For species distribution data, five field surveys were conducted in the upper reaches of the Dadu River - Minjiang River between July 2022 and May 2024. Latitude and longitude coordinates of *A. victorialis* were recorded using a GPS positioning system. To avoid model overfitting caused by clustered distribution points, a 1-km buffer was created around each point using the buffer tool in ArcGIS 10.2, retaining only one point per 1-km buffer. This process yielded 79 valid sample points, which were imported into Microsoft Excel and saved as a CSV file. The distribution records of *A. victorialis* are shown in **Figure 1b**.

The study incorporated a total of 41 environmental variables, including 19 bioclimatic factors, 16 soil factors, 3 topographic factors, 1 Human Footprint factor, 1 Landuse factor, and 1 NDVI factor. Both modern and future climate data were downloaded from the WorldClim database (http://worldclim.org/data/index.html, download time: August 2023, original resolution: 1 km²). Three scenarios (SSP126, SSP245, and SSP585) from the future climate system scenario models were applied, representing low, medium, and high greenhouse gas emission scenarios, respectively. Soil and topographic factor data were sourced from the Harmonized World Soil Database (HWSD) of the Food and Agriculture Organization of the United Nations (http://www.fao.org/faostat/en/#data, download time: August 2023, original resolution: 1 km²). Human Footprint data were obtained from the 2009 Human Footprint dataset, provided by the NASA Socioeconomic Data and Applications Center (SEDAC), which utilizes eight variables: built environments, population density, power infrastructure, croplands, pastures, roads, railways, and navigable waterways (Original resolution: 1 km²). The Normalized Difference Vegetation Index (NDVI) is the difference between near-infrared and red reflectance values, provided by the Land Processes Distributed Active Archive Center (LPDAAC) at the U.S. Geological Survey (USGS) Earth Resources Observation and Science (EROS) Center (http://LPDAAC.usgs.gov, download time: August 2023, original resolution: 250m). Land use data were derived from the Resource and Environment Data Cloud Platform of the Chinese Academy of Sciences (http://www.resdc.cn/Default.aspx, download time: August 2023, original resolution: 1 km²). The spatial resolution of all factors was set to 1 km² to ensure consistency in environmental data.

To standardize the spatial resolution of all environmental variables to 1 km², we used ArcGIS Pro software to resample data with an original resolution other than 1 km² (e.g., NDVI). For data with an original resolution of 1 km² (e.g., climate, soil, topography, human footprint, and land use), they were directly used or subjected to necessary unit conversions. Ultimately, the spatial resolution of all environmental factors was uniformly set to 1 km².

To avoid overfitting in model predictions caused by collinearity among environmental variables, the R language was used for VIF (Variance Inflation Factor) variable screening, PCA (Principal Component Analysis) verification, and Spearman correlation tests. These steps aimed to improve the accuracy of the ecological niche model by reducing model complexity (Elith et al., 2006). The process consisted of four main steps: (1) Preliminary correlation screening: To mitigate the impact of multicollinearity on model accuracy, Spearman rank correlation analysis was first performed on all candidate environmental variables. Variables with a correlation coefficient threshold of |r|< 0.7 were retained (**Supplementary Figures S1, S2**). (2) Handling highly correlated variables: For variable pairs with a Spearman correlation coefficient |r| ≥ 0.7, the variable with greater ecological significance or relevance to the research objective was retained, while its redundant counterpart was excluded. (3) Multicollinearity test: After the initial Spearman screening, the Variance Inflation Factor (VIF) was calculated for the remaining variables. Variables with a VIF ≥ 5, indicating severe multicollinearity, were removed. (4) PCA verification: Principal Component Analysis (PCA) was conducted on the variables retained after the previous two steps. PCA results were used to visualize and validate the independence and data structure of the screened variables, ensuring they effectively represented the main environmental variations while minimizing redundancy. This process yielded 16 final environmental variables (**Supplementary Table S1**).

### 2.3 Integrated model construction and implementation

The integrated model (Ensemble) constructed via weighted averaging outperformed all single models in accuracy (**Figure 1f**). The biomod2 package was used to develop an integrated model, requiring species presence data and pseudo-absence data. A total of 1290 pseudo-absence points were randomly generated using the “random” method within the study area. Data were split into a training set (75%) and a validation set (25%) using biomod2*_tuning* for parameter optimization (Barbet-Massin et al., 2012). With equal weights for presence and pseudo-absence data, 100 simulated models were generated via 10 repetitions. Only models with an actual skill statistic (TSS) ≥ 0.7 were retained in the final integrated model using weighted averaging.

Following established methodologies (Huang et al., 2025a, 2025b), the built-in bm_FindOptimStat function was used to convert continuous values into binary values based on thresholds, thereby obtaining optimal scores for the given evaluation. When using the BIOMOD_EnsembleForecasting prediction function, the parameter metric.binary was set to “TSS” to generate binary maps in the results. A 0/1 threshold (Cutoff = 0.351) was derived from the model results: areas below this threshold were classified as non-suitable habitats, while areas above the threshold were divided into three suitability classes: Lowly suitable region (0.351–0.567), Moderately suitable region (0.567–0.784), and Highly suitable region (0.784–1). The Distribution Change Between Binary SDMs tool in the SDM Tools plugin for ArcGIS was used to calculate changes in niche area across different periods.

**Figure 2:**
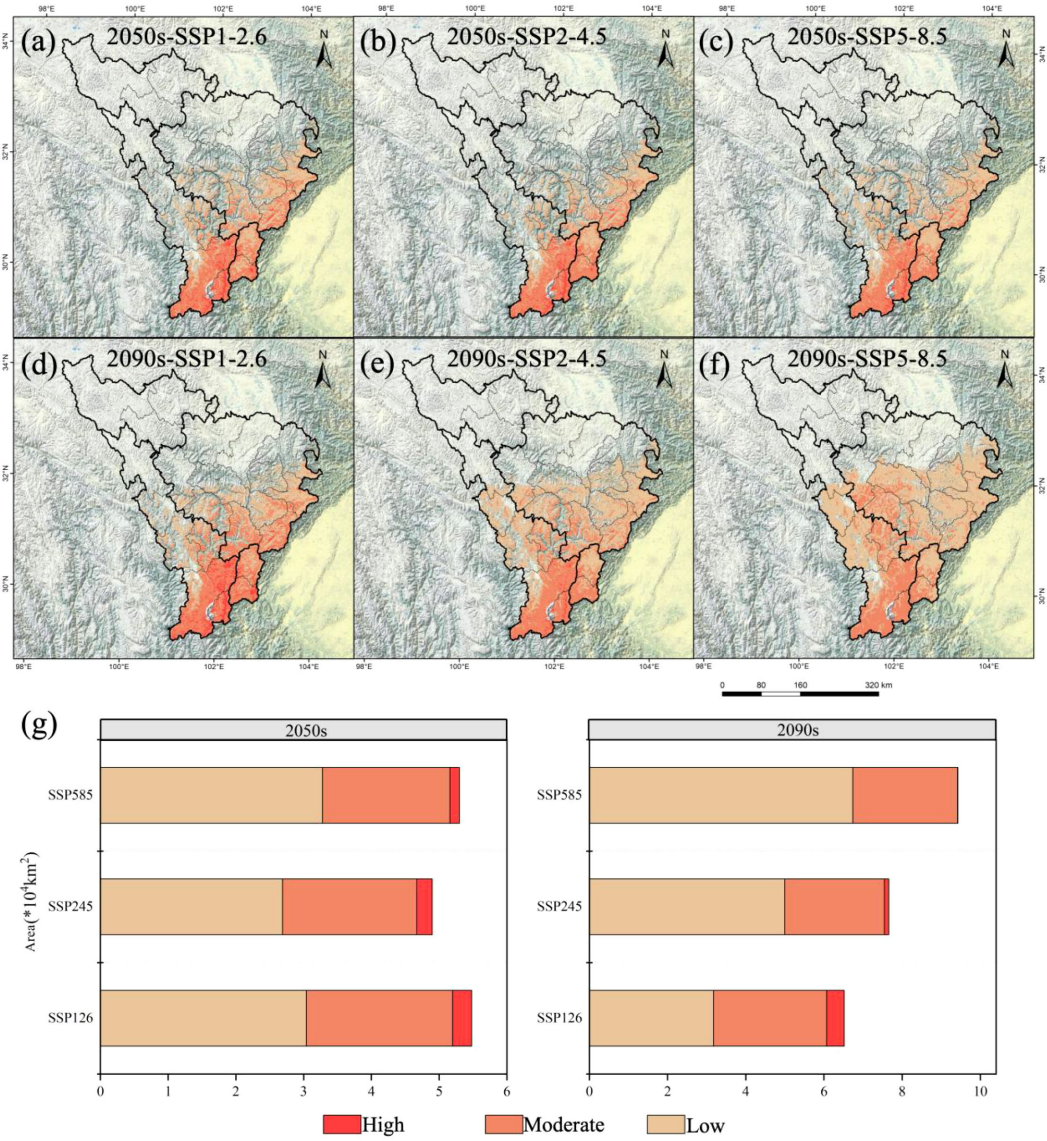
Potential geographical distribution of *A. victorialis* in the upper reaches of the Dadu River - Minjiang River under future climate change scenarios. Panels **(a–c)** show distributions under SSP1-2.6, SSP2-4.5, and SSP5-8.5 scenarios for the 2050s, respectively; **(d–f)** depict distributions for the 2090s under the same scenarios. Panel **(g)** illustrates the three-level suitability zones of *A. victorialis* under the three climate scenarios for both periods.

**Figure 3:**
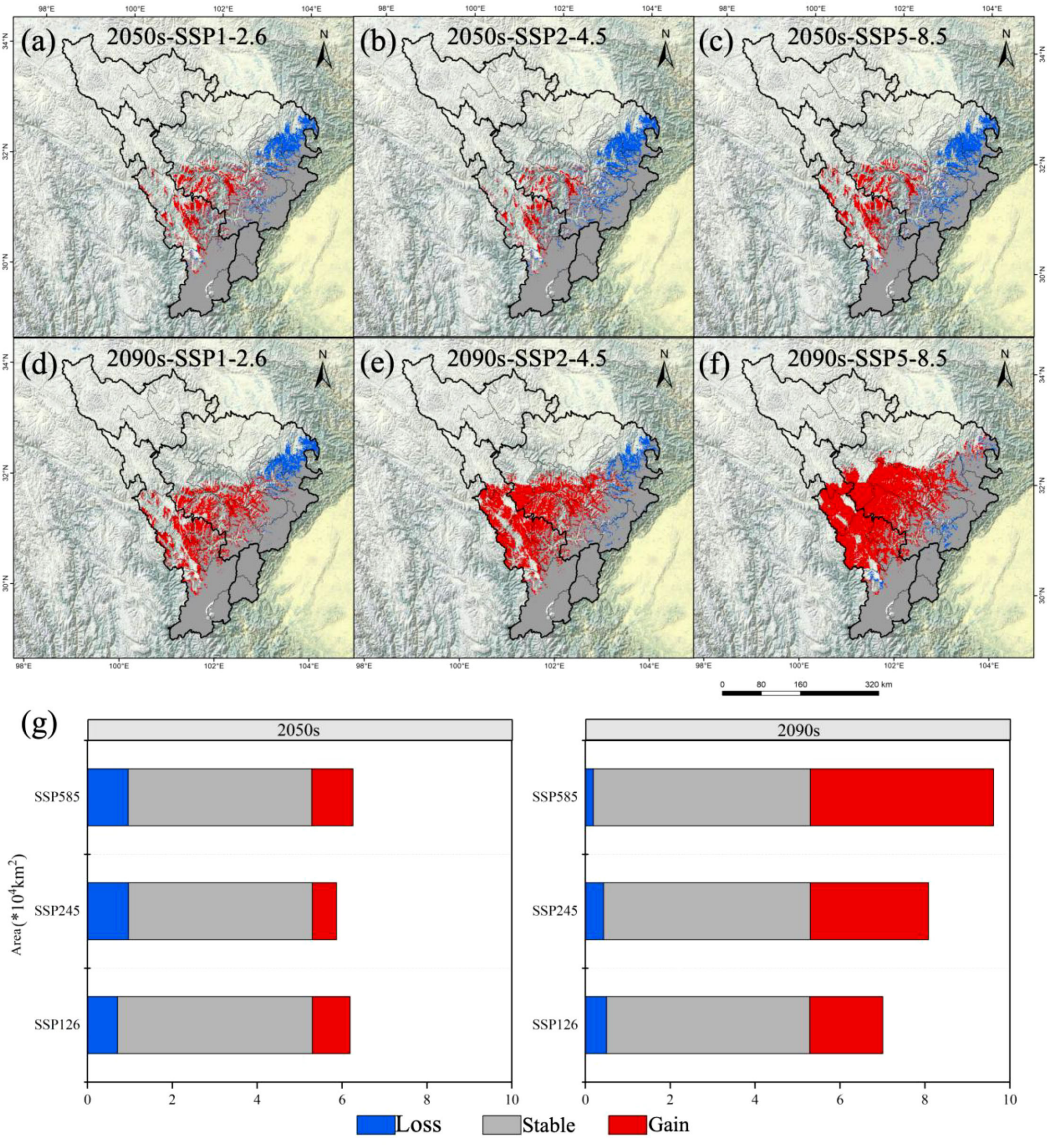
Changes in the potential geographical distribution of *A. victorialis* in the upper reaches of the Dadu River - Minjiang River under future climate change scenarios. Panels **(a–c)** and **(d–f)** show distribution changes under SSP1-2.6, SSP2-4.5, and SSP5-8.5 for the 2050s and 2090s, respectively. Panel **(g)** quantifies changes in suitable habitat area under the three scenarios for both time periods.

Building upon this, our study employed an ensemble modeling approach to predict the potential geographic distribution of *A. victorialis* in the upper reaches of the Dadu River - Minjiang River under three emission scenarios (SSP1-2.6, SSP2-4.5, and SSP5-8.5) for the current period, 2050, and 2090. This enabled us to quantify the areal extent of its potential distributional ranges in response to future climate change scenarios (**Figure 2**). Additionally, we projected the potential future distributions of *A. victorialis* (in 2050 and 2090) under the same climate scenarios using the ensemble model. Spatial overlay analysis was then performed in ArcGIS to visualize shifts in the potential distribution of this critical wild forage resource in the upper Dadu River -Minjiang River basin under changing climatic conditions (**Figure 3**).

### 2.4 Evaluation of species distribution models

Species distribution models (SDMs) often overestimate or underestimate distributions (false positives/negatives). To assess accuracy, three standard metrics were used: the receiver operating characteristic curve area (AUC), the true skill statistic (TSS), and the Kappa statistic (Kappa) (Jiang et al., 2023; Yang et al., 2024a). Evaluation criteria for these metrics are provided in **Supplementary Table S2**.

### 2.5 Selection of dominant environmental variables

Cumulative contribution rates were used to identify dominant environmental drivers, with the top three contributing variables defined as key factors—consistent with prevailing methodologies in the literature (Huang et al., 2022; Yang et al., 2022a; Liu et al., 2024b, 2024a).

### 2.6 Analysis of multivariate environmental similarity surfaces and most dissimilar variables

For MESS and MOD analysis, a dual validation framework was employed to assess the impacts of climate change on potential distributions (Zhao et al., 2021a, 2021b; Wen et al., 2022). The MESS algorithm quantifies deviations between future climate scenarios and baseline (current suitable habitat) conditions, where a similarity index (S) of 100 indicates perfect climate matching, a value of 0< S< 100 reflects climatic differences, with lower S values indicating a higher degree of climate anomaly, and S ≤ 0 signifies extreme climate anomalies beyond baseline ranges (Elith et al., 2010; Geange et al., 2011; Zhao et al., 2020). MOD analysis, a natural extension of MESS, was used to identify the individual variables that contribute most to environmental dissimilarity. When the S-value of a grid cell is< 0, MOD explicitly indicates which variable(s) exceed the calibration range of the model (Elith et al., 2010).

In this study, we conducted MESS and MOD analyses based on the contribution rates of environmental variables used in the ensemble modeling. Following established protocols (Huang et al., 2025b), environmental variables with contribution rates greater than 1.5% were selected to construct the multivariate reference baseline. The analytical workflow was implemented using the density.tools. Novel module integrated within Maxent.jar, with spatial visualization performed in ArcGIS. According to the variable contribution rates derived from the ensemble model (**Supplementary Table S2**), eight variables exceeded the 1.5% threshold: annual mean temperature (bio1), temperature seasonality (bio4), maximum temperature of the warmest month (bio5), minimum temperature of the coldest month (bio6), temperature annual range (bio7), annual precipitation (bio12), precipitation of the driest month (bio14), and gravel content (t_gravel). These variables were used for dual validation via MESS and MOD analyses to assess the risks of extrapolation and environmental novelty.

### 2.7 Niche change and centroid migration patterns

To quantify niche differentiation and its environmental drivers for *A. victorialis*, we used the ecospat package to calculate niche overlap rates under current and future climate scenarios, visualize niche changes, and compute the niche parameter D (observed value, range 0–1), where 0 indicates no overlap and 1 indicates complete overlap, to assess climate change impacts on its ecological niche (Levins, 2020). Niche breadth in geographic and environmental spaces was calculated as the average Levins’ B1 (inverse concentration) and B2 (uncertainty) values, with values approaching 0 indicating narrower niches and values approaching 1 indicating wider niches (Warren et al., 2010; Yue et al., 2011).

Regarding the migration patterns of the centroid of the total suitable area, the SDMTool package (v1.1-21) in R was used to compute the centroid coordinates of suitable habitats for *A. victorialis* across different periods and climate scenarios (Equation 1). Latitude/longitude coordinates, as well as migration distances between centroids, were calculated in ArcGIS, with the results visualized to depict centroid migration trends.


(1) $$\left(X_{C},\;Y_{C}\right)=\frac{{{(}^{\sum }w_{i}x_{i}}^{\sum }}{w_{i}}\frac{{{,}^{\sum }w_{i}y_{i}}^{\sum }}{w_{i}})$$

Here, *w_i_* represents the suitability value of grid cells, and *x_i_*, *y_i_* are geographic coordinates. Migration vectors were constructed using current and future centroid coordinates to analyze migration direction (azimuth angle) and distance (km).

### 2.8 Establishing the relationship between cultivation productivity and environmental suitability

Nutritional components (routine nutrients, bioactive substances, and amino acid profiles) of *A. victorialis* were measured at 36 randomly selected distribution points using food science methods (**Supplementary Table S3**). Referring to previous research methods (Feng et al., 2023; Huang et al., 2025a), we established a cultivation productivity evaluation model based on the synergistic effects of ecological suitability and nutritional components (Equation 2), defined as:


(2) P=S+N

Ecological suitability (S): Derived from species distribution model output (presence probability values), with suitability data extracted via spatial interpolation and weighted according to model reliability. Nutritional components (N): Indicator weights were determined using the entropy weight method (details on nutrient types, weight settings, and rationales are provided in Text S1). After standardizing indicators (**Supplementary Table S4**), weighted summation was performed using Equation 3:


(3) $$N=\sum_{i=1}^{4}w_{i}\cdot X_{i}^{norm}$$

Model validation was conducted using the ggtrendline package in R (v4.1.2), fitting seven nonlinear regression models (**Table 1**) to quantify the relationship between cultivation productivity and ecological suitability (Sun et al., 2021). The optimal model (ΔAIC< 2) was selected based on the Akaike Information Criterion (AIC) to construct a suitability-productivity model. Using the optimal model, the distribution of potential cultivation and production areas of *A. victorialis* under current and future climatic conditions was predicted.

**Table 1:** Seven types of models used for modeling the relationship between productivity and suitability.

Model code	Model type
exp2P model	y=a*exp(b*x)
exp3P model	y=a*exp(b*x)+c
line2P model	y = a*x + b
line2P model	y=a*x^^^2+b*x+c
line2P model	y=a*ln(x)+b
line2P model	y=a*x^^^b
line2P model	y=a*x^^^b+c

Additionally, based on the suitability-productivity model and the relationship between suitability and productivity of *A. victorialis*, productivity was categorized into three grades: high productivity (> 0.57), moderate productivity (0.37–0.57), and low productivity (< 0.37). In this study, the potential cultivation zones of *A. victorialis* were delineated into three levels, where high productivity areas were classified as first-level cultivation zones, moderate productivity areas as second-level cultivation zones, and low productivity areas as third-level cultivation zones (Sun et al., 2021).

## 3 Results

### 3.1 Model simulation accuracy validation

Simulation results of *A. victorialis* from various models showed that, overall, the suitable habitats of *A. victorialis* are mainly concentrated in the eastern, southern, and central parts of the upper reaches of the Dadu River - Minjiang River region. Although all models predicted a consistent general trend, there were significant differences in the specific results among different models. The model parameters were optimized using the ‘biomod_tuning’ function, with parameter checks conducted in each iteration based on selected metrics (AUC, Kappa, or TSS). Among single models, Random Forest (RF) and Maxent were the most effective for predicting the potential spatial distribution of key wild edible plant resources in the upper Dadu River - Minjiang River region, while Generalized Additive Models (GAM), Artificial Neural Networks (ANN), and Surface Range Envelope (SRE) performed the worst (**Figure 1f**). Evaluation of the Ensemble model for *A. victorialis* yielded a TSS of 0.854, AUC of 0.999, and Kappa of 0.958 (**Figure 1f**). Using valid evaluation metrics to assess model accuracy is a critical step in determining model reliability and applicability. According to the criteria for AUC, TSS, and Kappa statistics (**Supplementary Table S2**), the Ensemble model exhibited the best fit and most ideal predictive performance.

### 3.2 Environmental factors influencing the potential distribution of *A. victorialis*

Among the environmental factors used in the integrated model, annual temperature range (Bio7, 50.63%), precipitation of the warmest quarter (Bio12, 10.44%), and temperature seasonality (Bio4, 9.21%) ranked top three in contribution, with a cumulative contribution rate of 70.28%, identifying them as the dominant drivers of *A. victorialis*’s potential distribution (**Supplementary Table S1**).

### 3.3 Analysis of multivariate environmental similarity surfaces and most dissimilar variables

**Figure 4:**
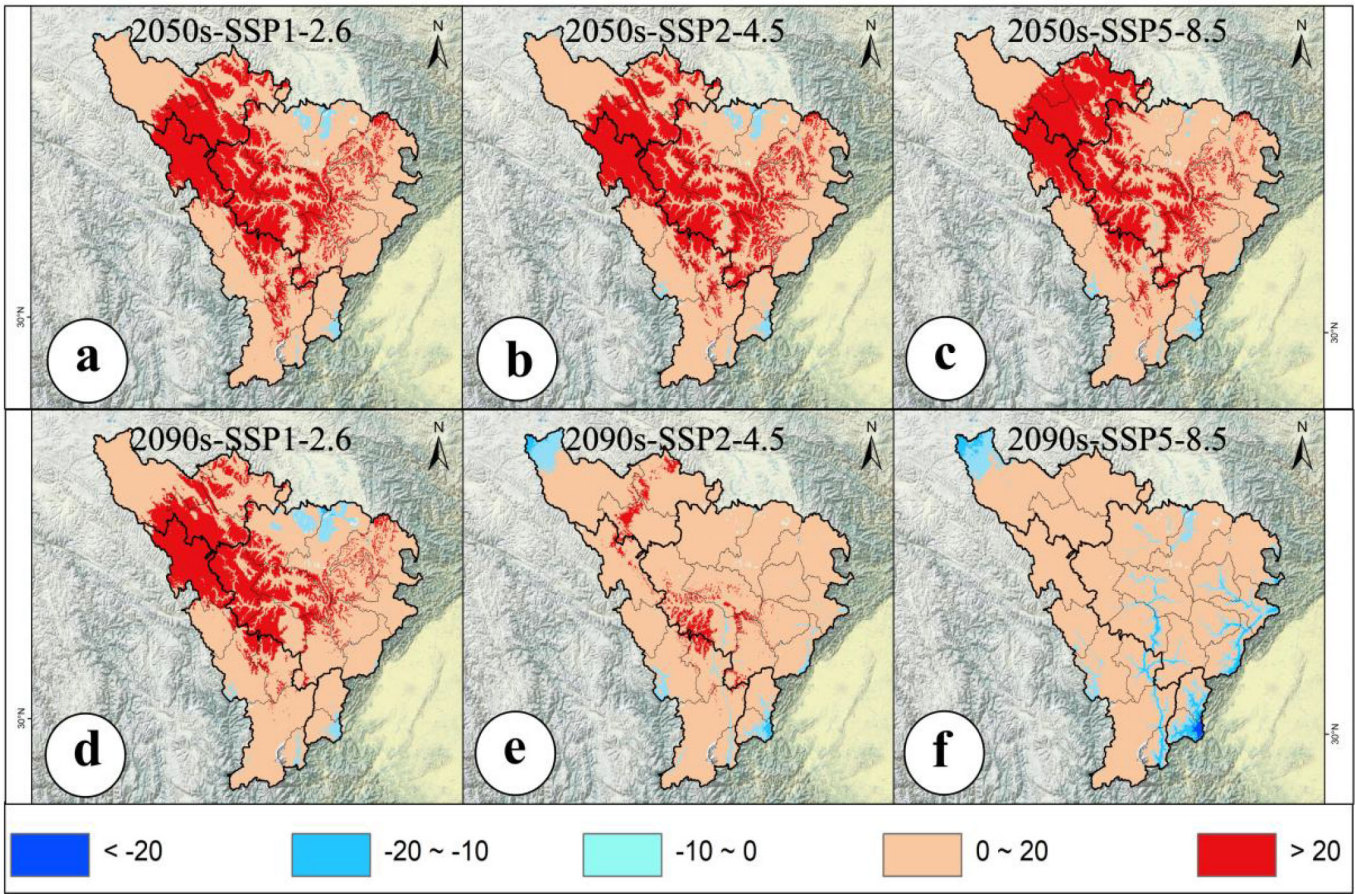
Analysis of MESS. **(a)** MESS for 2050s-SSP1-2.6, **(b)** MESS for 2050s-SSP2-4.5, **(c)** MESS for 2050s-SSP5-8.5, **(d)** MESS for 2090s-SSP1-2.6, **(e)** MESS for 2090s-SSP2-4.5, **(f)** MESS for 2090s-SSP5-8.5.

**Figure 5:**
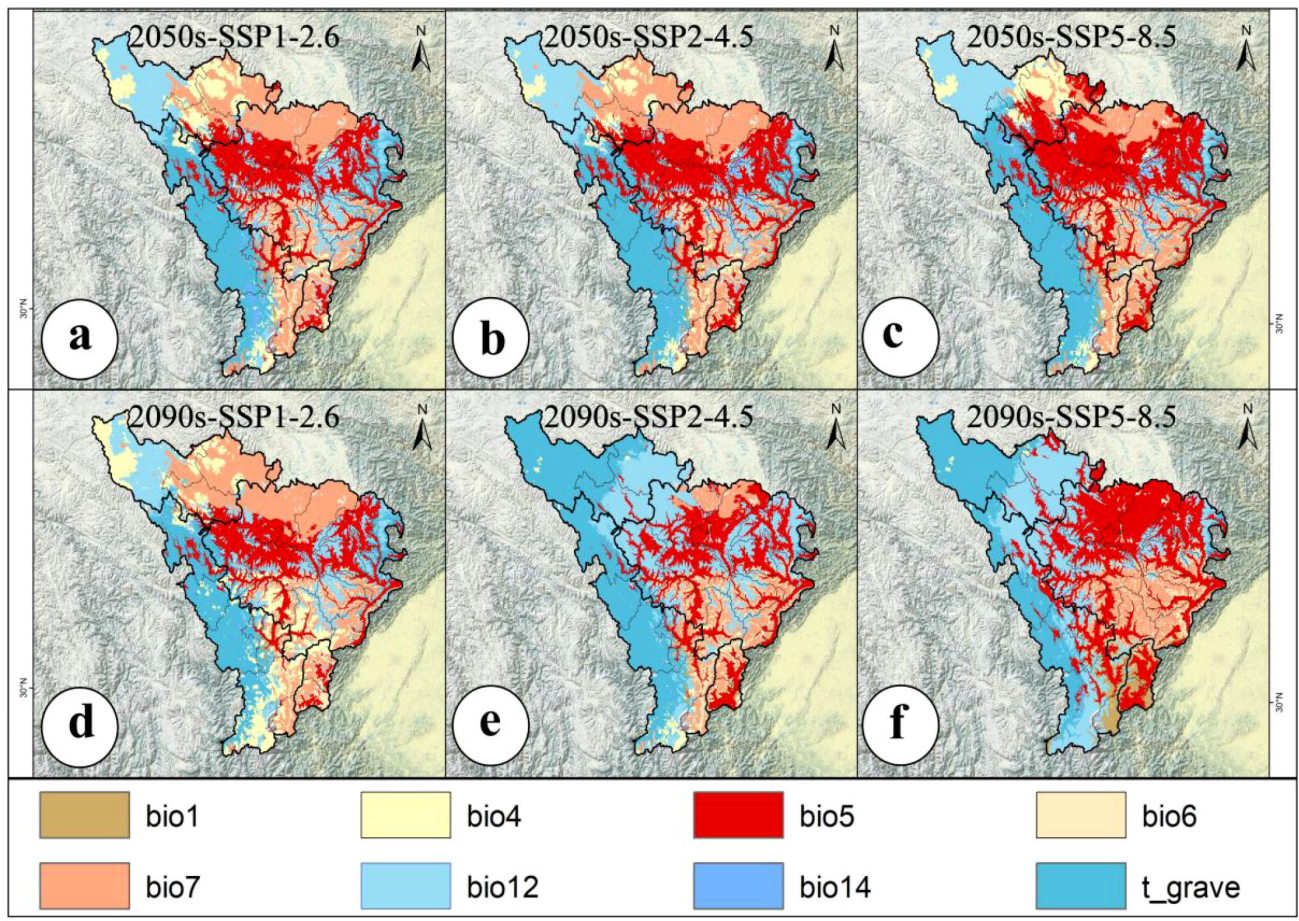
Analysis of MOD. **(a)** MOD for 2050s-SSP1-2.6, **(b)** MOD for 2050s-SSP2-4.5, **(c)** MOD for 2050s-SSP5-8.5, **(d)** MOD for 2090s-SSP1-2.6, **(e)** MOD for 2090s-SSP2-4.5, **(f)** MOD for 2090s-SSP5-8.5.

Under different climate scenarios, the average multivariate similarity values for the 79 modern distribution points of *A. victorialis* were 9.41, 8.27, 7.66, 5.93, 5.90, and 1.67 for mid-century and end-of-century periods, respectively (**Figure 4**). The lowest MESS value in the SSP5-8.5 (high-emission) scenario in 2090 indicated that intensified climate warming may restructure the water-heat regime (e.g., extreme precipitation or expanded annual temperature increases), thereby weakening ecosystem stability (**Figure 4**). In contrast, higher MESS values under SSP1-2.6 (sustainable development) suggested that low-carbon pathways help maintain environmental similarity (**Figure 4**). Based on the MOD analysis, three environmental variables, namely temperature annual range (Bio7), precipitation of the warmest quarter (Bio18), and maximum temperature of the warmest month (Bio5), exhibited significant anomalies under different climate change scenarios for the mid-century and end-century periods (**Figure 5**). Among these three most dissimilar variables, two were identified as the dominant environmental factors influencing the potential geographic distribution of *A. victorialis* according to their contribution rates (**Supplementary Table S1**). Collectively, significant differences in MESS and MOD in the upper Dadu River - Minjiang River region indicate a concerning level of climate anomaly, highlighting substantial future vulnerability of *A. victorialis* to climate change.

### 3.3 Potential distribution areas of *A. victorialis* under climate change and temporal variations

The integrated model simulated the potential geographic distribution of *A. victorialis* under current climate conditions (**Figure 1c**). The total suitable habitat area in the upper Dadu River - Minjiang River region was 5.30×10^4^km², with high-suitability habitats accounting for 0.72×10^4^km² (13.58% of the total). These high-suitability areas were primarily distributed in patchy and clustered patterns in Kangding City, Luding County, and Tianquan County, with scattered occurrences in Baoxing, Wenchuan, and Mao Counties (**Figure 1c**). Medium-suitability habitats covered 2.52×10^4^km² (47.54% of the total), surrounding high-suitability zones and forming clustered distributions in Songpan, Heishui, Mao, Xiaojin, and Lixian Counties (**Figure 1c**).

The potential geographic distribution and changes of *A. victorialis* in the upper reaches of the Dadu River - Minjiang River under three climate scenarios over two future periods are described as follows. The total suitable habitat area increased most significantly under SSP5-8.5 in 2090 (77.92% increase, 4.13×10^4^km²), while the most significant decrease occurred under SSP2-4.5 in 2050 (7.55% decrease, 0.40×10^4^km²) (**Figure 2**). High-suitability habitats experienced the most severe decline under SSP5-8.5 in 2090 (98.61% decrease, 0.71×10^4^km²), with no increases observed in any scenario; the smallest drop occurred under SSP1-2.6 in 2090 (37.50% decrease, 0.27×10^4^km²) (**Figure 2**). Medium-suitability habitats showed the most significant increase under SSP1-2.6 in 2090 (14.68% increase, 0.37×10^4^km²) and the most significant decrease under SSP2-4.5 in 2050 (21.43% decrease, 0.54×10^4^km²) (**Figure 2**). Low-suitability habitats exhibited the most pronounced expansion under SSP5-8.5 in 2090 (227.18% increase, 4.68×10^4^km²), with no declines in any scenario; the smallest increase occurred under SSP2-4.5 in 2050 (30.58% increase, 0.63×10^4^km²) (**Figure 2**). It can be seen that against the backdrop of climate warming, the suitability of *A. victorialis* does not show a simple linear change. Instead, a polarized pattern emerges, characterized by an increase in moderately and lowly suitable habitats and a decrease in highly suitable habitats. This trend is particularly pronounced under the SSP585 scenario.

The total suitable habitat area of *A. victorialis* exhibited an expanding trend compared to the current period. Under the SSP5-8.5 scenario, the largest expansion occurred in the 2050s (18.26% increase, 0.97×10^4^km²), while the smallest expansion occurred under SSP2-4.5 (10.78% increase, 0.57×10^4^km²) (**Figure 3**). In the 2090s, SSP5-8.5 again drove the most significant expansion (81.64% increase, 4.32×10^4^km²), with SSP1-2.6 showing the smallest increase (32.53% increase, 1.72×10^4^km²) (**Figure 3**). These results highlight that high-emission scenarios (SSP5-8.5) most strongly drive range expansion of *A. victorialis.*

### 3.4 Niche change and habitat centroid migration trajectory of *A. victorialis* in future periods

We quantified niche differentiation and environmental drivers of *A. victorialis* under three emission scenarios (SSP1-2.6, SSP2-4.5, SSP5-8.5) for 2050 and 2090. Using the ecospat package, niche overlap rates were calculated and visualized between the current climate and future scenarios, based on distribution points and climate data. Centroid coordinates of suitable habitats under current and future climates were used to reveal migration trajectories and trends of potential suitable habitats.

**Figure 6:**
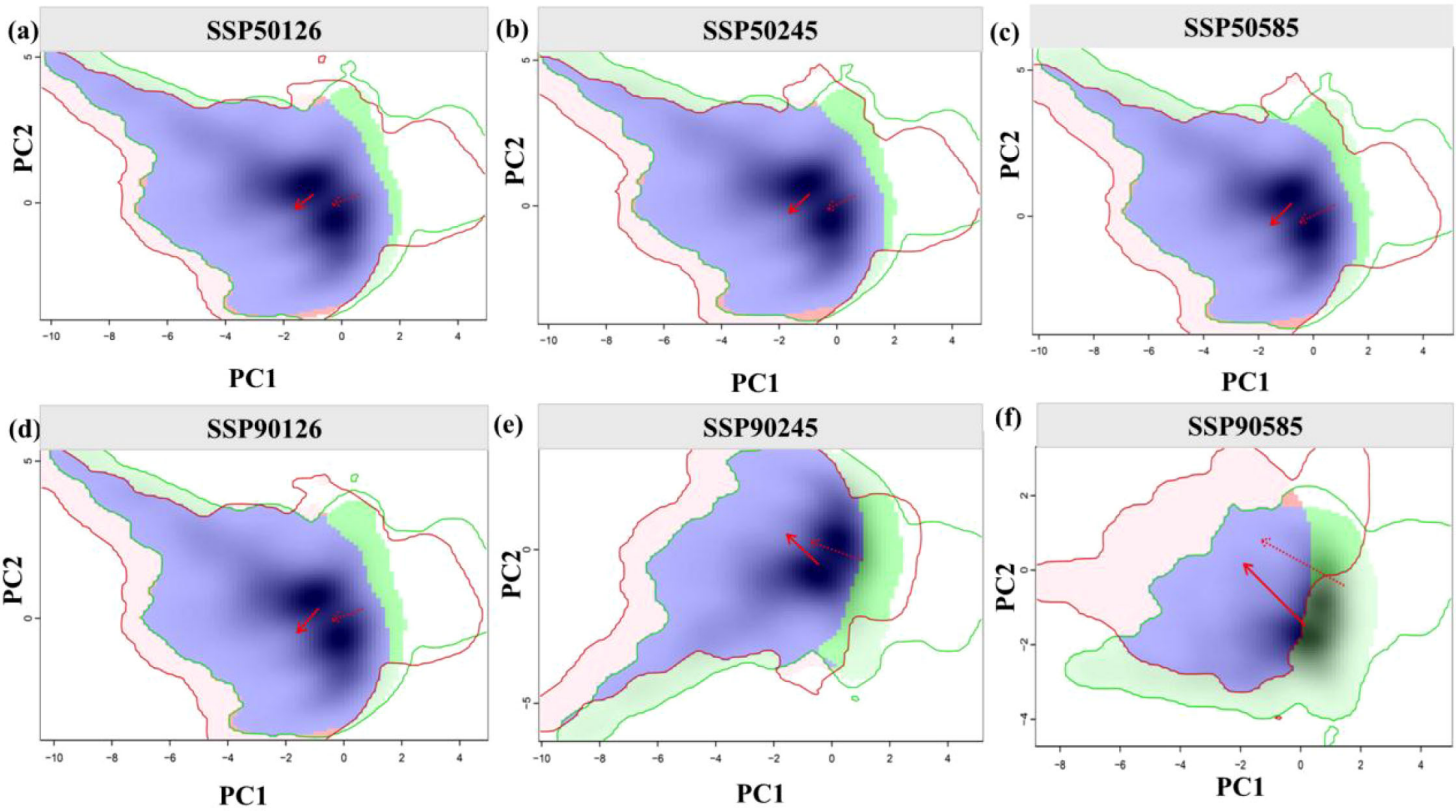
Ecological niche changes of *A. victorialis* under climate change. Panels **(a–c)** and **(d–f)** depict niche changes under SSP1-2.6, SSP2-4.5, and SSP5-8.5 for the 2050s and 2090s, respectively. Green and red shadows represent species occurrence density in current and future scenarios, with blue indicating overlap. Solid and dashed lines denote 100% and 50% of available environmental space, respectively. The red arrow marks the migration of the climate niche centroid (solid line) and background range centroid (dashed line) between periods.

Niche overlap of *A. victorialis* in the upper Dadu River - Minjiang River is shown in **Figure 6**. Niche overlap rates remained relatively high under SSP1-2.6 (D = 0.696 in the 2050s, D = 0.659 in the 2090s) but decreased significantly under SSP5-8.5 (D = 0.603 in the 2050s, D = 0.502 in the 2090s), indicating niche specialization with climate warming and potential migration to higher elevations or latitudes. Priority should be given to protecting high-elevation core distribution areas. Principal component analysis (PCA) showed that the first two components explained 68.67–71.64% of environmental variance (PC1: 51.34–53.08%; PC2: 17.33–18.56%), with annual temperature range, precipitation of the warmest quarter, and isothermality as the main drivers of niche changes. The future climatic niche centroid will shift toward areas with higher annual temperature range and warm-season precipitation.

**Figure 7:**
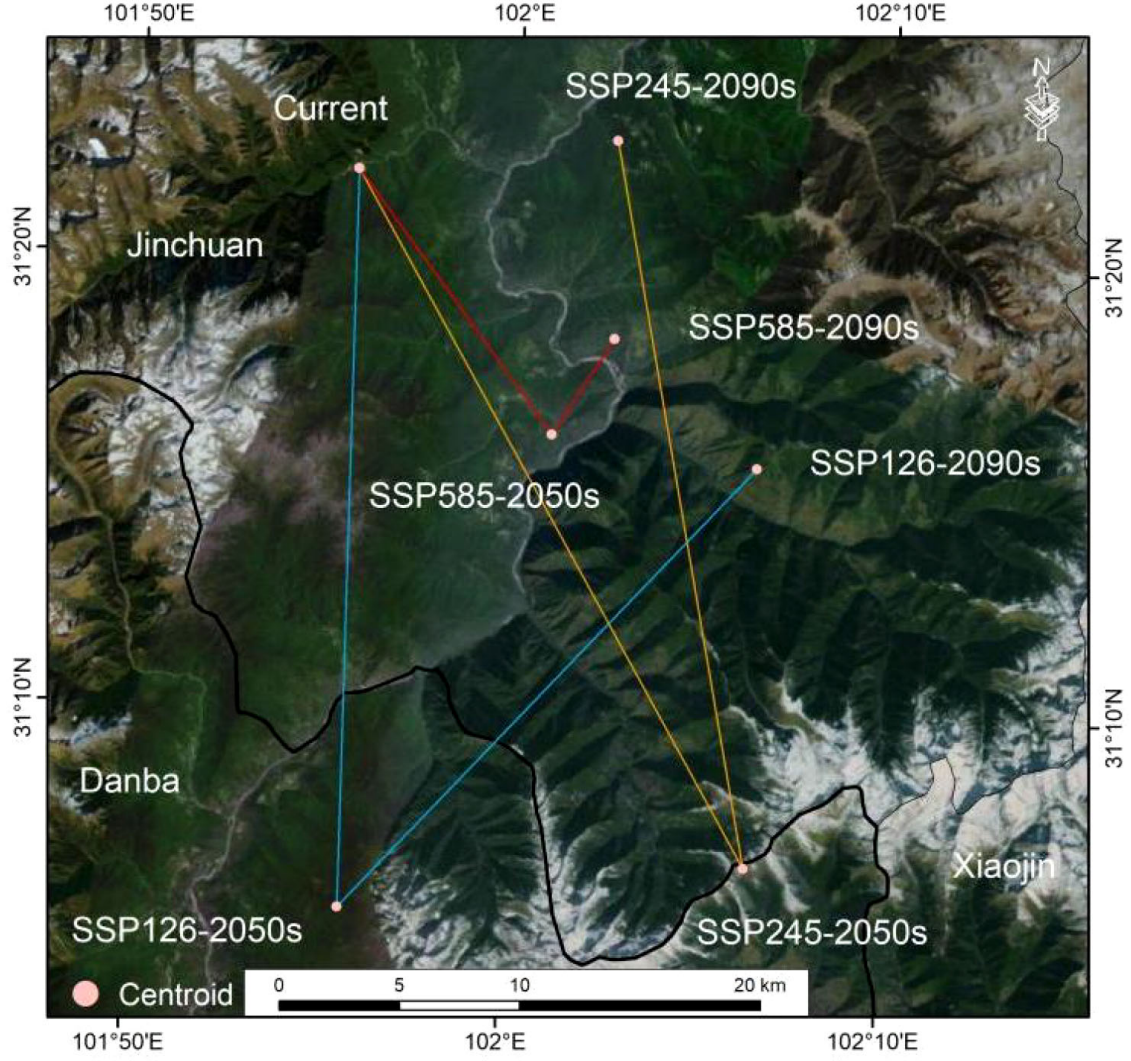
Variations in the centroids of suitable areas of *A. victorialis* under climate change scenarios.

The centroid of current suitable habitats for *A. victorialis* in the upper Dadu River - Minjiang River is located at 101.9285°E/31.3662°N (**Figure 7**). Under SSP1-2.6, the centroid first moved 3.03 km southwest to (101.9285°E, 31.0926°N) in the 2050s, then 25.22 km northeast to (102.1087°E, 31.2590°N) in the 2090s (**Figure 7**). Under SSP2-4.5, it shifted 33.01 km southeast to (102.1075°E, 31.1111°N) in the 2050s, then 30.31 km northwest to (102.0428°E, 31.3789°N) in the 2090s (**Figure 7**). Under SSP5-8.5, the centroid moved 13.62 km southeast to (102.0174°E, 31.2696°N) in the 2050s, then 47.31 km northeast to (102.0440°E, 31.3056°N) in the 2090s (**Figure 7**). Overall, across all three scenarios, the centroid of suitable habitats first shifted to the low-latitude southeast from the baseline period to 2050, then migrated toward higher latitudes and the northwest by 2090.

### 3.5 Dynamics of potential cultivation areas for *A. victorialis* in different periods

Based on the Akaike Information Criterion (AIC), among the seven linear models, there was a significant positive correlation between habitat suitability and productivity of *A. victorialis*. The exp2P model, with an R² of 0.942, was the optimal model in this study, followed by the exp3P model (R² = 0.940), the line3P model (R² = 0.939), the power3P model (R² = 0.935), the log2P model (R² = 0.929), the line2P model (R² = 0.808), and finally the line2P model (R² = 0.726) with the poorest performance. Therefore, the line3P model was selected in this study to construct the productivity-suitability model.

**Figure 8:**
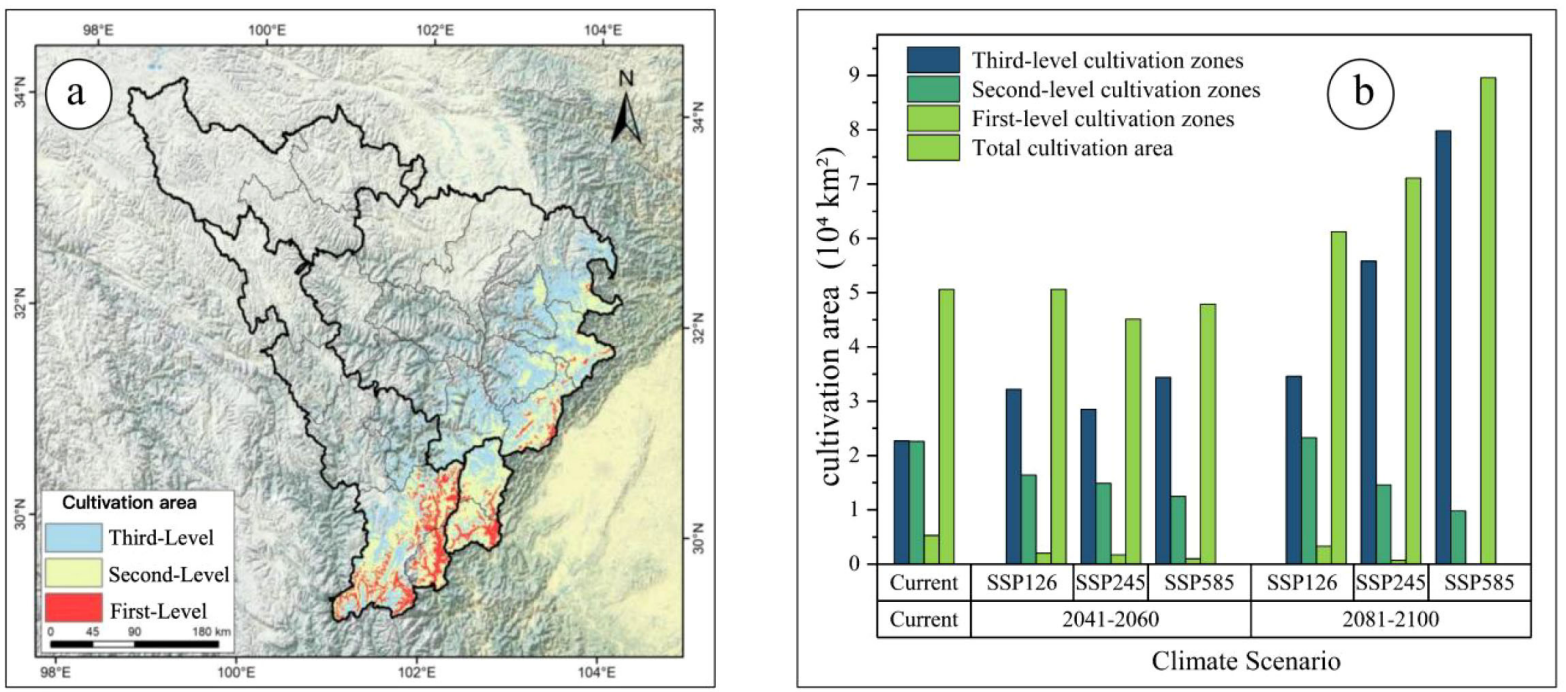
**(a)** Current cultivation areas of *A*. *victorialis*; **(b)** Changes in cultivation area across different periods.

Based on the suitability-productivity model, productivity was classified into three tiers: high, medium, and low. High-productivity areas correspond to first-level cultivation zones, medium-productivity areas to second-level zones, and low-productivity areas to third-level zones. Under current climate conditions, the total suitable cultivation area is 5.06×10^4^km², including 0.53×10^4^km² of first-level zones, 2.26×10^4^km² of second-level zones, and 2.27×10^4^km² of third-level zones (**Figure 8b**).

**Figure 9:**
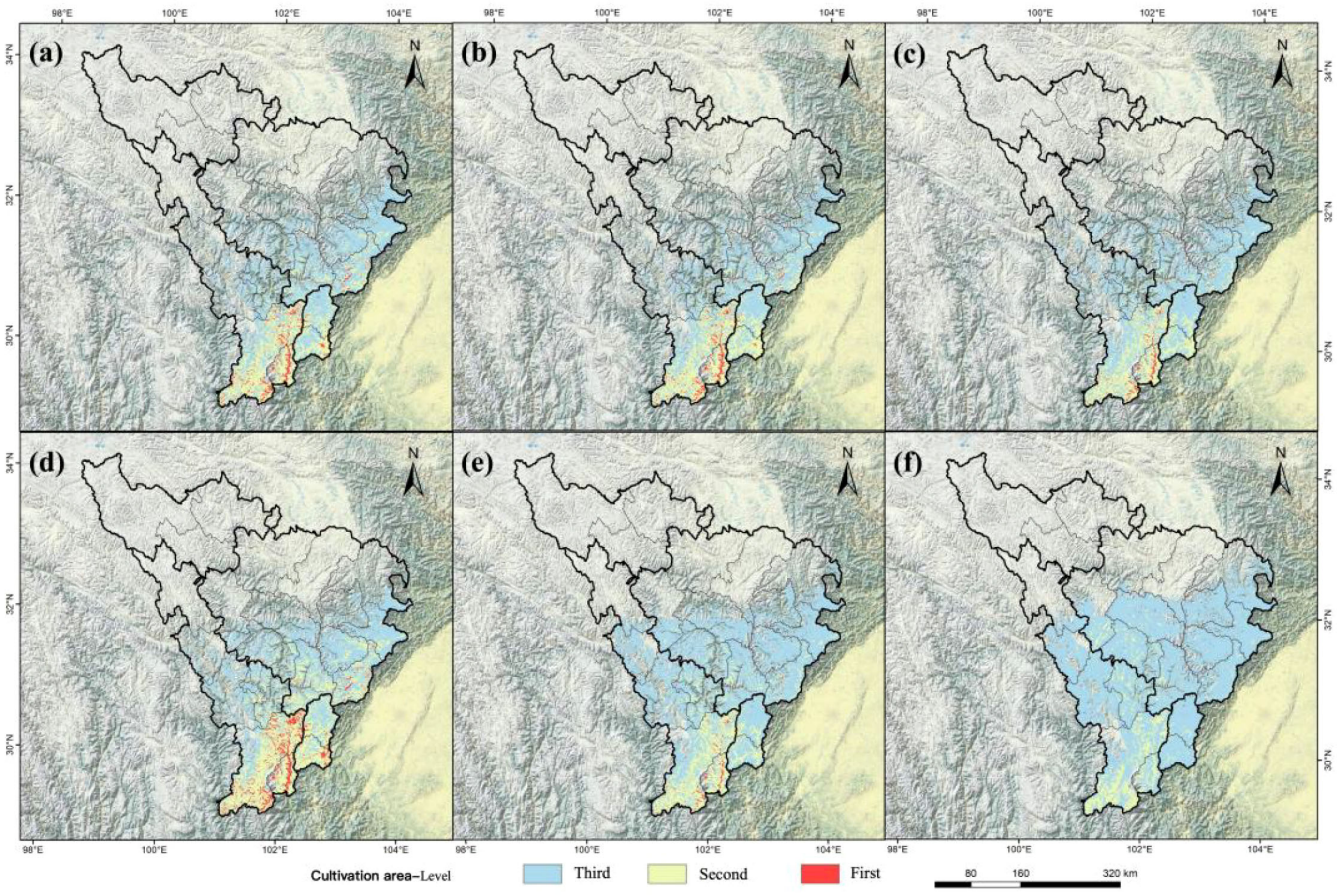
Three-level cultivation zones of *A. victorialis* in the upper Dadu River - Minjiang River under future climate scenarios. Panels **(a–c)** and **(d–f)** depict zones under SSP1-2.6, SSP2-4.5, and SSP5-8.5 for the 2050s and 2090s, respectively.

Cultivation areas are primarily concentrated in low-altitude regions of the upper Min and Dadu Rivers (**Figure 8a**). Quantitative analysis of spatiotemporal changes in cultivation areas under different Shared Socioeconomic Pathways (SSPs) revealed pronounced shifts in first- and second-level zones compared to the current period. Projections for the two future periods indicate that losses in first-level zones and second-level zones will intensify over time and with increasing severity of the emission scenario (**Figure 9**).

Under all three scenarios and both periods, first- and second-level zones experienced varying degrees of loss, with the most drastic declines under SSP5-8.5 (**Figure 9**). Third-level zones expanded into the central regions of the upper Dadu River - Minjiang River. In the 2050s under SSP5-8.5, first-level cultivation areas shrank to 0.17×10^4^km², and the second-level regions to 1.25×10^4^km² (**Figure 9**). By the 2090s under SSP5-8.5, first-level zones were lost entirely, and second-level areas decreased to 0.98×10^4^km² (**Figure 9**), indicating significant degradation of cultivation areas under high-emission scenarios. These changes also reflect a northward shift in cultivation zones, driven by rising temperatures and altered precipitation patterns.

## 4 Discussion

### 4.1 Significance of exploring the variation patterns of potential distribution and cultivation areas of *A. victorialis* in the upper reaches of the Dadu River - Minjiang River

*A. victorialis* is an important wild vegetable utilized by residents in the study area, boasting multiple values in terms of edibility, medicinal use, and economy. Field surveys show that the annual collection of *A. victorialis* in the upper reaches of the Minjiang River can reach 200–300 tons, generating an average annual income of 1,200–1,500 yuan per household. Building on this, unlike previous studies that commonly employed a single SDMs, the present study systematically compared and screened 12 common SDMs in the region to identify the one best suited to the complex topographic and climatic characteristics of the study area (Zhao et al., 2021a; Huang et al., 2025a). On this basis, SDMs and the area suitability-productivity model were applied to explore the variation patterns of the potential distribution areas and cultivation areas of *A. victorialis* under climate change, providing a more comprehensive and scientific decision-making basis for the sustainable utilization of *A. victorialis* resources.

### 4.2 Factors influencing model accuracy and the accuracy of the ensemble model

SDMs predict potential distributions by correlating species occurrence data with environmental variables; however, their prediction accuracy is highly dependent on factors such as model construction, algorithm selection, data quality, and sample size. In particular, insufficient sample size can significantly degrade model performance and increase uncertainty, making it difficult to fully capture complex niche dimensions (Elith and Leathwick, 2009; Pecchi et al., 2019; Liu et al., 2024a; Naimi et al., 2014; Xie et al., 2023). Thus, large datasets can better describe complex relationships and interactions. In some cases, models that perform well with large sample sizes do not necessarily perform well with small sample sizes (Natale et al., 2013). This issue requires an investigation into potential trade-offs between sample size and model complexity. Furthermore, Thuiller et al. (2009) argued that the Biomod2 platform effectively addresses model bias caused by sample spatial aggregation through cross-validation and parameter tuning. Elith et al. (2010) noted that ensemble models can mitigate the sensitivity of single models to specific environmental variables and reduce the risk of overfitting through weighted average strategies.

This study included 79 valid distribution records. The accuracy of the ensemble model was evaluated using AUC values, TSS values, and Kappa statistics, all of which reached an “excellent” level. These results indicate that the Biomod2 ensemble model used in this study achieved good performance in exploring the potentially suitable habitat distribution of *A. victorialis* in the upper reaches of the Dadu River - Minjiang River under climate change, and the results are reliable.

### 4.3 Impacts of climate change on *A. victorialis*

The rapid climate change in the Anthropocene has exerted a profound impact on many species within regional ecosystems (Wan et al., 2024; Ramirez-Barahona et al., 2025; Carosi et al., 2020). Regarding the niche changes of *A. victorialis*, the dynamic changes in its ecological niche showed that the degree of niche overlap in all pairwise comparisons between the current and future scenarios decreased with the increasing intensity of climate change. Among the environmental factors limiting *A. victorialis* in this study, temperature annual range (Bio7, 50.63%), precipitation of the warmest quarter (Bio12, 10.44%), and temperature seasonality (Bio4, 9.21%) ranked top three in contribution rate, with a cumulative contribution rate of 70.28%, making them the dominant environmental factors affecting the potential geographic distribution of *A. victorialis*. These findings align with Feng Bo (Feng, 2023), who identified extreme temperature sensitivity as a key constraint on *A. victorialis* growth and quality, consistent with the collapse of high-suitability habitats observed here. (Hideaki et al., 2009). and (Kanazawa et al., 1997). further corroborated the critical role of temperature in regulating bulb dormancy and seedling germination, respectively. Precipitation impacts, highlighted by Feng Bo (Feng, 2023), align with broader regional studies showing climate variables as primary distribution drivers (Liu et al., 2022, 2025; Yang et al., 2022b; Zhang et al., 2024).

Based on environmental factors under three emission scenarios for 2050 and 2090, combined with modern climate conditions, this study predicted the potential geographic distribution of *A. victorialis* under these scenarios. The results showed that under climate change, the potential highly suitable habitats and Grade I cultivation areas of *A. victorialis* in the upper reaches of the Dadu River - Minjiang River generally showed a decreasing trend, while the lowly suitable areas and Grade III cultivation areas showed an increasing trend. A study by (Pauli et al., 2012). indicated that under climate warming, some plants face the “mountaintop trap” effect in alpine ecosystems. It can be seen that *A. victorialis* in alpine regions also faces the “mountaintop trap” effect. A study by (Thomas et al., 2004). on the risk of biological extinction in sample areas covering 20% of the Earth’s surface showed that the impact of climate warming on the potential geographic distribution of species is bidirectional, and not all species are at risk of extinction or benefit from climate change. Obviously, *A. victorialis* in alpine ecosystems, under climate warming and affected by the “mountaintop trap” effect, does not show a simple linear change in suitability. Instead, a polarized pattern emerges: moderately and lowly suitable habitats and lower-quality cultivation areas increase, while highly suitable habitats and high-quality cultivation areas decrease.

Under climate change, the phenomenon of plant habitat centroids migrating to higher latitudes or elevations has been confirmed by numerous classic studies. Parmesan and Yohe (2003), integrating global species distribution data, found that 80% of species showed a trend of migrating to polar regions or higher elevations due to climate warming, with temperate plants responding particularly significantly to temperature changes. This pattern has been verified in multiple ecosystems across Eurasia. (Chen et al., 2011). showed that the northern boundary of *Fagus sylvatica* distribution expanded toward the Scandinavian Peninsula at a rate of 30–50 km per decade, and its driving mechanism was directly related to the weakening of winter low-temperature constraints in high-latitude regions. Similarly, Lenoir et al (Lenoir et al., 2008), in their analysis of 171 plant species in six major European mountain ranges, showed that the average elevation of plant communities increased by 29 meters per decade over the past century, which was highly synchronized with the regional warming rate. In the study area, (Huang et al., 2025c). explored the variation patterns of potentially suitable habitats of *P. aquilinum* var. *latiusculum* in the upper reaches of the Dadu River - Minjiang River under climate change, and found that the migration rate of *P. aquilinum* var. *latiusculum* was much higher than that of low-elevation species, with a significant northward shift in its niche. The impact of climate warming on the potential geographic distribution of species is mainly manifested in the shift of potential geographic distribution areas to higher latitudes or elevations, as well as the expansion and contraction of potential geographic distribution areas (Garcia et al., 2013). In this study, the trend of potentially suitable habitats of *A. victorialis* shifting to higher latitudes and northeastern regions under future climate change scenarios conforms to this characteristic, which is consistent with the “climate-driven-niche shift” mechanism revealed by the above classic cases, further supporting the latitudinal adaptation strategy of Northern Hemisphere plants in response to climate warming.

The upper reaches of the Dadu River - Minjiang River are a transition zone between forests and grasslands on the Qinghai-Tibet Plateau (Huang et al., 2025b; Zhang, 2018). This study showed that the niche of *A. victorialis* has a trend of migrating from the current forest-grassland transition zone to high-elevation grasslands, with a much greater migration amplitude under high-emission scenarios than under low-emission scenarios. The stability of grassland ecosystems is much lower than that of forest and shrub ecosystems (Sun et al., 2021). Large-scale exploitation and utilization of *A. victorialis* in grassland ecosystems will undoubtedly pose a huge challenge to the ecological stability and diversity of grasslands. Therefore, in future exploitation and utilization of *A. victorialis* in the upper reaches of the Dadu River - Minjiang River, the possibility of its migration to high-elevation grassland areas under climate change needs to be considered.

### 4.4 Adaptive management strategies for *A. victorialis* in response to climate change

To address this challenge, this study proposes a three-level response strategy of “core area protection—alternative habitat development—adaptive cultivation”: ecological red lines are delineated in the existing Grade I cultivation areas in Wenchuan and Lixian County to limit the collection intensity of *A. victorialis*; adaptive cultivation technologies are developed in Grade II cultivation areas, using the “terrace supplementary lighting + sunshade net” technology to control the annual temperature range within 22°C, and using drip irrigation systems to compensate for precipitation deficits; when species cannot keep up with climate change through natural diffusion, assisted migration is an effective method to mitigate the impacts of climate change (McLachlan et al., 2007). This study suggests that alternative habitat development can be implemented in Songpan County, the target area of centroid migration.

Based on the unique climatic conditions in the upper reaches of the Dadu River - Minjiang River, this study puts forward three suggestions for the sustainable utilization of wild vegetables such as *A. victorialis*: (1) Enhance awareness and promote rational development. It is necessary to further strengthen the general survey of wild vegetable resources such as *A. victorialis*, understand their efficacy, clarify their distribution areas and growth patterns, investigate their production, and adhere to the principle of equal emphasis on utilization and protection. (2) Strengthen research and realize scientific utilization. To meet development needs, it is necessary to increase systematic research. Key technologies for the artificial cultivation of wild vegetables such as *A. victorialis* should be studied to increase commodity quantities and continuously meet the growing consumption needs of residents. (3) It is recommended to give priority to standardized cultivation in the current core cultivation areas to meet market demand, and at the same time, adopt adaptive management strategies with dynamic adjustment of cultivation areas.

### 4.5 Research limitations and prospects

This study provides a macro-planning basis under climate change scenarios for the sustainable management of *A. victorialis* resources in high mountain gorge area, but there are still several aspects that need to be further refined. Firstly, although the Human Footprint index was incorporated into the modeling, its contribution rate was not fully quantified due to the spatial characteristic of generally low intensity of human activities in the study area. In the future, it is urgent to integrate high spatiotemporal resolution dynamic data of human activities and construct a “climate-human” dual-driving model to accurately assess the harvesting pressure and land use competition effects in hotspots such as Wenchuan County and Kangding City. Secondly, it is necessary to systematically analyze the synergistic effects of multi-dimensional influencing mechanisms: coupling land use change models to simulate habitat fragmentation processes, embedding socioeconomic driving factors at the community level, and quantifying the competition coefficients between *A. victorialis* and associated plants through field control experiments to improve niche dynamic prediction. In addition, sample size also has a significant impact on the accuracy of model predictions, and more comprehensive sample data are required to optimize the prediction results. Beyond that, more accurate evaluation methods need to be added. The current evaluation methods may all produce false positives; for example, field evaluation can be added to assess the accuracy of prediction results. Given the inherent unpredictability of future variables, practical applications must comprehensively evaluate these confounding factors within adaptive management frameworks.

## 5 Conclusions

This study revealed the evolution patterns of the distribution and cultivation areas of *A. victorialis* in the upper reaches of the Dadu River-Minjiang River under climate change by integrating species distribution models and dynamic cultivation productivity models. It quantitatively evaluated the potential limiting environmental factors, potential distribution areas, and cultivation areas of *A. victorialis* in this region under climate change, and clarified the ecological requirements of *A. victorialis*. The results showed that the current highly suitable habitats and core cultivation areas of *A. victorialis* are primarily distributed in a fragmented manner in low-altitude river valley regions, including Kangding City, Luding County, Tianquan County, Baoxing County, Wenchuan County, and Mao County. Against the backdrop of climate warming, both the potential distribution areas and cultivation areas of *A. victorialis* in the upper reaches of the Dadu River - Minjiang River exhibit a polarized pattern of “high-quality contraction and medium-low quality expansion”, with a concurrent gradual shift in its climatic niche. For the utilization of *A. victorialis* under climate warming, on the one hand, standardized cultivation in the current core cultivation areas can meet market demand; on the other hand, adaptive management strategies should be adopted to adjust cultivation areas dynamically. In summary, this study not only provides decision support for the sustainable utilization of *A. victorialis* resources but also offers a reference for biodiversity conservation and livelihood adaptation in the high mountain gorge area under global change.

## Data Availability

The original contributions presented in the study are included in the article/**Supplementary Material**. Further inquiries can be directed to the corresponding author/s.
