# Supplementary material for: Changes in potential distribution and cultivation areas of Allium victorialis L. under climate change
Source: Front Plant Sci. 2025 Sep 3;16:1629527. doi: 10.3389/fpls.2025.1629527 (PMC12442045; doi:10.3389/fpls.2025.1629527)
Supplement: Supplementary file 1 [file DataSheet1.docx]

**Supplementary material for**

Changes in Potential Distribution and Cultivation Areas of *Allium victorialis* L. Under Climate Change

**Text S1: Nutritional Component Types, Weight Allocation, and Rationale for *Allium victorialis* L.**

This study measured the routine nutritional components, bioactive substances, and amino acid profiles of *Allium victorialis* L. After several discussions with experts from the Sichuan Provincial Key Laboratory of Ecology and the Sichuan Provincial Engineering Center for Disaster Prevention and Mitigation, weights were assigned to each component based on their nutritional functions, health values, and environmental sensitivity.

A total weight of 100% was allocated as follows:

- **Routine nutrients and bioactive components (75% total weight):** Protein (12%): Assigned the highest weight among routine nutrients as a core indicator of growth and quality, directly regulated by nitrogen use efficiency. Dietary fiber (10%): Reflects soil and moisture conditions through cell wall composition and supports gut health, justifying its weight. Fat (7%), crude fiber (5%), and ash (1%): Allocated sequentially based on energy density, stress resistance, and mineral content, respectively. Starch (15%): A key carbohydrate metabolite regulated by light-temperature conditions via enzymes like ADP-glucose pyrophosphorylase, critical for carbon metabolism. Total flavonoids (12%): Linked to enhanced antioxidant capacity induced by UV-B radiation through the phenylpropanoid pathway. Total saponins (8%) and tannins (5%): Represent immune potential and stress response, respectively, guiding their weight allocation.
- **Amino acid profiles (25% total weight):** **Essential amino acids (12%):** Each of the 8 essential amino acids was assigned 1.5%, reflecting their dependency on nitrogen metabolism efficiency. Conditionally essential amino acids (5%): Arginine (2%): Involved in salt stress response. Cysteine (1.5%): Indicates sulfur metabolism levels. Tyrosine (1.5%): Signifies secondary metabolism regulation capacity. Glutamate (4%): As a nitrogen metabolism hub, critical for salt stress regulation. Other non-essential amino acids (4%): Proline (2%): Contributes to drought response via osmotic regulation. Remaining amino acids (0.5% each): Minor roles in metabolic processes.


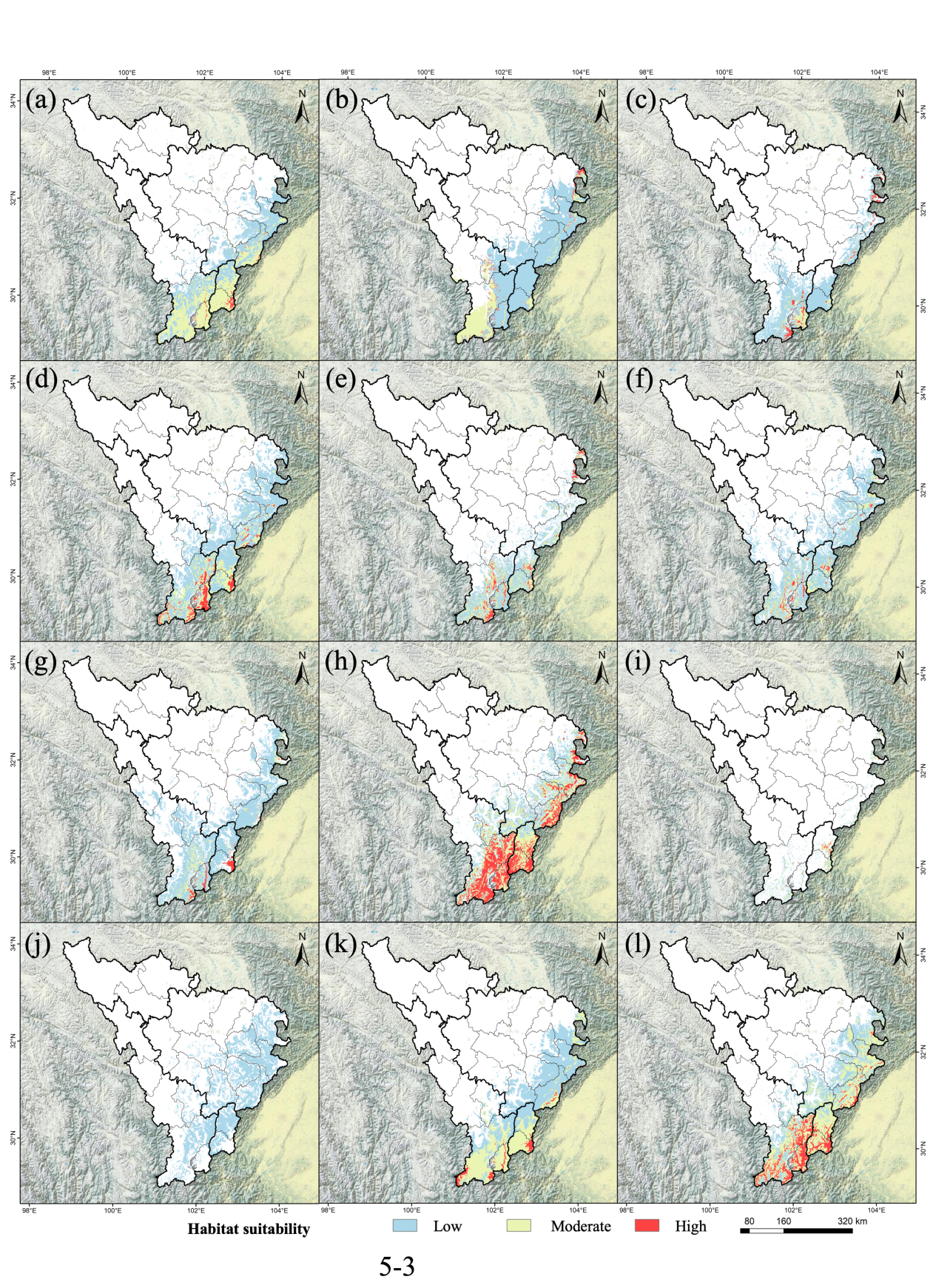


**FIGURE S1.** Potential distribution of *A. victorialis* in the upper reaches of the Dadu-Minjiang River predicted by multiple models. (a) ANN model, (b) GTA model, (c) FDA model, (d) GAM model, (e) GBM model, (f) GLM model, (g) MARS model, (h) Maxent model, (i) RF model, (j) SER model, (k) XGBOOST model, (l) Ensemble model.


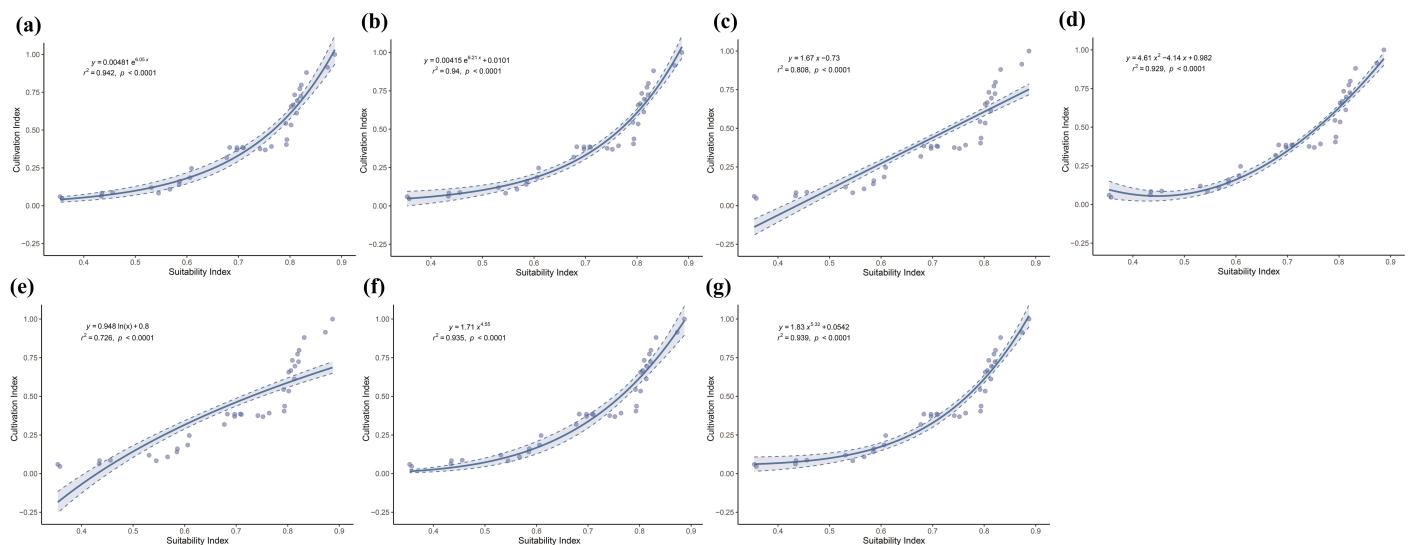


**FIGURE S2.** The relationship between suitability and productivity of *A. victorialis*

**TABLE S1.** Sources of modeling environment variables.

| **Environment Variable** | **Source** | **Download URL** |
| --- | --- | --- |
| Current climate data | WorldClim database | [http://worldclim.org/data/index.html](http://worldclim.org/data/index.html) |
| Future climate data | WorldClim database | [http://worldclim.org/data/index.html](http://worldclim.org/data/index.html) |
| Soil factors | Food and Agriculture Organization (FAO) World Soil Database | [http://www.fao.org/faostat/en/#data](http://www.fao.org/faostat/en/#data) |
| Topographic factors | Food and Agriculture Organization (FAO) World Soil Database | [http://www.fao.org/faostat/en/#data](http://www.fao.org/faostat/en/#data) |
| Human Footprint data | NASA Socioeconomic Data and Applications Center (SEDAC) | / |
| Normalized Difference Vegetation Index (NDVI) | U.S. Geological Survey (USGS) Land Processes Distributed Active Archive Center (LPDAAC) | [http://lpdaac.usgs.gov](http://lpdaac.usgs.gov/) |
| Land use data | Data Center for Resources and Environmental Sciences, Chinese Academy of Sciences | [http://www.resdc.cn/Default.aspx](http://www.resdc.cn/Default.aspx) |

**TABLE S2.** Sixteen environmental variables included in the modeling.

| **Environment Variable** | **Abbreviation** | **Unit** | **Contribution Rate (%)** |
| --- | --- | --- | --- |
| Maximum temperature of warmest month | bio5 | °C | 8.4 |
| Minimum temperature of coldest month | bio6 | °C | 4.9 |
| Elevation | elev | m | 6.62 |
| Human Footprint | footprint | gha | 0.33 |
| Land cover | landcover | / | 0.13 |
| Seasonal dry matter production | dmps | g/m²/season | 0.01 |
| Gross primary productivity | gpp | g C/m²/year | 1.46 |
| Annual mean temperature | bio1 | °C | 3.48 |
| Annual precipitation | bio12 | mm | 10.44 |
| Temperature seasonality (coefficient of variation) | bio4 | C of V | 9.21 |
| Annual temperature range | bio7 | °C | 50.63 |
| Precipitation of driest month | bio14 | mm | 0.02 |
| Precipitation seasonality (coefficient of variation) | bio15 | C of V | 1.68 |
| Gravel content | t_gravel | - | 1.52 |
| Soil organic carbon content | t_oc | % | 1.16 |
| Soil pH (water) | t_ph_h2o | / | 0.01 |

**TABLE S3.** Relationship between evaluation indicators (ROC, TSS, Kappa) and model accuracy.

| **Evaluation Indicators** | **Fail** | **Differ from** | **General** | **Good** | **Excellent** |
| --- | --- | --- | --- | --- | --- |
| AUC | 0.50–0.60 | 0.60–0.70 | 0.70–0.80 | 0.80–0.90 | 0.90–1.00 |
| TSS | 0.00–0.40 | 0.40–0.55 | 0.55–0.70 | 0.70–0.85 | 0.85–1.00 |
| Kappa | 0.00–0.40 | 0.40–0.55 | 0.55–0.70 | 0.70–0.85 | 0.85–1.00 |

**TABLE S4:** Standardized results of various indicators for *A. victorialis*

|  |  |  | **Conventional nutritional components** | | | | | | **Active substance components** | | | **Amino acid components** | | | | | | | | | | | | | | | | |
| --- | --- | --- | --- | --- | --- | --- | --- | --- | --- | --- | --- | --- | --- | --- | --- | --- | --- | --- | --- | --- | --- | --- | --- | --- | --- | --- | --- | --- |
| Longitude | Latitude | *S*uitability | CFT | CFR | CAH | CPN | DFR | STH | SAN | FLD | TAN | ASP | THR | SER | GLU | GLY | ALA | PRO | CYS | VAL | MET | ILE | LEU | TYR | PHE | HIS | LYS | ARG |
| 102.189 | 29.829 | 0.887 | 7.000 | 5.000 | 1.000 | 12.000 | 10.000 | 15.000 | 8.000 | 12.000 | 5.000 | 0.500 | 1.500 | 0.500 | 4.000 | 0.500 | 0.500 | 2.000 | 1.500 | 1.500 | 1.500 | 1.500 | 1.500 | 1.500 | 1.500 | 1.500 | 1.500 | 2.000 |
| 102.089 | 30.054 | 0.873 | 6.682 | 4.677 | 0.950 | 11.226 | 9.773 | 14.500 | 7.652 | 11.053 | 4.750 | 0.453 | 1.313 | 0.442 | 3.667 | 0.444 | 0.462 | 1.667 | 1.125 | 1.327 | 0.857 | 1.091 | 1.157 | 1.125 | 1.200 | 1.200 | 1.167 | 1.500 |
| 103.873 | 32.407 | 0.832 | 6.745 | 4.748 | 0.943 | 11.512 | 9.605 | 14.200 | 7.304 | 10.421 | 4.250 | 0.423 | 1.225 | 0.406 | 3.453 | 0.426 | 0.412 | 1.587 | 0.713 | 1.281 | 0.557 | 0.995 | 1.071 | 0.990 | 1.158 | 1.080 | 1.097 | 1.430 |
| 101.951 | 30.387 | 0.822 | 6.364 | 4.355 | 0.900 | 10.065 | 9.318 | 13.500 | 6.957 | 9.789 | 4.500 | 0.358 | 0.875 | 0.288 | 2.667 | 0.296 | 0.308 | 1.133 | 0.750 | 0.981 | 0.429 | 0.682 | 0.771 | 0.750 | 0.840 | 0.900 | 0.833 | 1.200 |
| 102.804 | 30.286 | 0.821 | 6.427 | 4.400 | 0.876 | 10.870 | 9.214 | 13.400 | 5.913 | 9.474 | 4.000 | 0.313 | 0.831 | 0.273 | 2.313 | 0.283 | 0.277 | 1.013 | 0.638 | 0.865 | 0.429 | 0.682 | 0.737 | 0.705 | 0.756 | 0.810 | 0.733 | 1.070 |
| 102.289 | 29.999 | 0.819 | 6.236 | 4.355 | 0.880 | 10.606 | 9.000 | 13.250 | 6.609 | 9.158 | 3.750 | 0.330 | 0.750 | 0.231 | 1.667 | 0.204 | 0.192 | 0.667 | 0.750 | 0.635 | 0.137 | 0.409 | 0.343 | 0.525 | 0.600 | 0.600 | 0.500 | 0.900 |
| 102.582 | 30.365 | 0.814 | 6.045 | 4.194 | 0.850 | 10.452 | 8.636 | 13.000 | 5.913 | 8.842 | 4.000 | 0.292 | 0.688 | 0.250 | 2.000 | 0.259 | 0.231 | 0.867 | 0.375 | 0.750 | 0.214 | 0.545 | 0.429 | 0.600 | 0.600 | 0.700 | 0.667 | 1.000 |
| 102.108 | 29.917 | 0.813 | 5.918 | 4.155 | 0.821 | 10.150 | 8.891 | 13.550 | 6.261 | 8.526 | 3.750 | 0.286 | 0.469 | 0.171 | 1.400 | 0.157 | 0.150 | 0.467 | 0.525 | 0.392 | 0.279 | 0.341 | 0.377 | 0.450 | 0.360 | 0.580 | 0.367 | 0.730 |
| 101.941 | 30.045 | 0.809 | 5.727 | 4.032 | 0.800 | 9.677 | 8.409 | 12.750 | 6.261 | 8.211 | 3.500 | 0.245 | 0.563 | 0.192 | 0.667 | 0.148 | 0.154 | 0.533 | 0.375 | 0.462 | 0.429 | 0.545 | 0.386 | 0.450 | 0.420 | 0.500 | 0.433 | 0.800 |
| 101.766 | 29.651 | 0.806 | 5.600 | 3.955 | 0.795 | 9.546 | 8.255 | 12.600 | 6.609 | 7.579 | 3.500 | 0.180 | 0.438 | 0.144 | 1.173 | 0.141 | 0.142 | 0.480 | 0.563 | 0.427 | 0.321 | 0.368 | 0.390 | 0.413 | 0.372 | 0.540 | 0.383 | 0.710 |
| 103.613 | 31.478 | 0.802 | 5.409 | 3.871 | 0.749 | 8.903 | 7.955 | 12.000 | 5.565 | 7.263 | 3.250 | 0.217 | 0.375 | 0.135 | 1.000 | 0.130 | 0.115 | 0.333 | 0.375 | 0.346 | 0.643 | 0.273 | 0.300 | 0.375 | 0.300 | 0.400 | 0.333 | 0.600 |
| 101.898 | 30.253 | 0.802 | 4.455 | 3.065 | 0.098 | 3.484 | 6.364 | 6.000 | 3.478 | 7.263 | 3.000 | 0.264 | 1.000 | 0.346 | 2.000 | 0.259 | 0.308 | 1.333 | 1.125 | 1.212 | 1.500 | 0.818 | 0.857 | 1.125 | 1.200 | 1.500 | 0.833 | 1.500 |
| 101.882 | 30.127 | 0.794 | 5.091 | 3.710 | 0.699 | 8.516 | 7.500 | 11.500 | 5.217 | 5.684 | 2.250 | 0.123 | 0.063 | 0.058 | 0.333 | 0.074 | 0.038 | 0.005 | 0.300 | 0.058 | 1.071 | 0.136 | 0.038 | 0.060 | 0.096 | 0.280 | 0.128 | 0.360 |
| 102.315 | 29.844 | 0.793 | 3.755 | 1.942 | 0.522 | 6.465 | 3.882 | 8.300 | 3.130 | 6.632 | 2.500 | 0.179 | 0.381 | 0.123 | 1.113 | 0.139 | 0.142 | 0.373 | 0.525 | 0.421 | 0.279 | 0.314 | 0.266 | 0.375 | 0.360 | 0.540 | 0.413 | 0.610 |
| 103.624 | 32.094 | 0.792 | 3.500 | 2.258 | 0.499 | 5.652 | 4.091 | 7.500 | 2.783 | 5.684 | 2.250 | 0.170 | 0.375 | 0.135 | 1.067 | 0.148 | 0.154 | 0.400 | 0.375 | 0.404 | 0.214 | 0.273 | 0.300 | 0.375 | 0.360 | 0.500 | 0.367 | 0.600 |
| 101.451 | 29.635 | 0.764 | 3.524 | 2.127 | 0.407 | 5.120 | 4.933 | 6.836 | 2.316 | 4.874 | 2.230 | 0.165 | 0.350 | 0.125 | 1.167 | 0.113 | 0.135 | 0.407 | 0.563 | 0.398 | 0.300 | 0.327 | 0.347 | 0.390 | 0.390 | 0.560 | 0.377 | 0.670 |
| 103.983 | 32.282 | 0.752 | 4.327 | 2.742 | 0.248 | 3.871 | 5.909 | 4.500 | 2.783 | 3.158 | 1.500 | 0.217 | 0.563 | 0.173 | 1.667 | 0.167 | 0.192 | 0.667 | 0.750 | 0.577 | 0.643 | 0.136 | 0.386 | 0.525 | 0.540 | 0.800 | 0.500 | 1.000 |
| 103.261 | 31.174 | 0.742 | 2.864 | 1.613 | 0.399 | 5.806 | 4.545 | 7.000 | 1.739 | 5.053 | 2.500 | 0.142 | 0.438 | 0.115 | 1.133 | 0.111 | 0.154 | 0.467 | 0.375 | 0.462 | 0.429 | 0.409 | 0.257 | 0.375 | 0.420 | 0.600 | 0.400 | 0.700 |
| 102.933 | 30.516 | 0.71 | 4.136 | 2.419 | 0.299 | 4.258 | 5.682 | 5.500 | 2.435 | 3.789 | 1.750 | 0.189 | 0.500 | 0.154 | 1.333 | 0.148 | 0.154 | 0.533 | 0.750 | 0.519 | 0.429 | 0.273 | 0.300 | 0.450 | 0.480 | 0.700 | 0.433 | 0.800 |
| 102.762 | 30.519 | 0.708 | 3.309 | 2.000 | 0.419 | 5.265 | 5.227 | 6.750 | 2.087 | 4.105 | 2.000 | 0.151 | 0.375 | 0.115 | 1.200 | 0.130 | 0.154 | 0.467 | 0.375 | 0.462 | 0.429 | 0.409 | 0.343 | 0.375 | 0.420 | 0.600 | 0.400 | 0.700 |
| 103.802 | 32.263 | 0.697 | 2.800 | 1.761 | 0.347 | 4.575 | 5.473 | 6.900 | 1.739 | 4.105 | 2.750 | 0.154 | 0.413 | 0.144 | 1.207 | 0.107 | 0.162 | 0.433 | 0.488 | 0.467 | 0.343 | 0.341 | 0.287 | 0.405 | 0.432 | 0.580 | 0.397 | 0.720 |
| 102.765 | 30.393 | 0.697 | 3.182 | 2.032 | 0.449 | 5.419 | 5.000 | 6.000 | 2.087 | 4.421 | 2.000 | 0.160 | 0.375 | 0.135 | 1.000 | 0.130 | 0.192 | 0.333 | 0.375 | 0.404 | 0.214 | 0.409 | 0.343 | 0.375 | 0.420 | 0.500 | 0.333 | 0.700 |
| 101.786 | 30.401 | 0.683 | 3.945 | 2.674 | 0.352 | 4.351 | 5.441 | 5.300 | 2.087 | 3.474 | 1.750 | 0.172 | 0.463 | 0.110 | 1.400 | 0.133 | 0.146 | 0.480 | 0.525 | 0.444 | 0.321 | 0.395 | 0.304 | 0.443 | 0.468 | 0.570 | 0.427 | 0.700 |
| 103.739 | 32.161 | 0.677 | 2.545 | 1.290 | 0.349 | 6.581 | 3.636 | 8.000 | 1.391 | 2.526 | 1.000 | 0.123 | 0.313 | 0.058 | 0.667 | 0.093 | 0.115 | 0.200 | 0.300 | 0.346 | 1.071 | 0.076 | 0.129 | 0.060 | 0.180 | 0.280 | 0.128 | 0.360 |
| 102.565 | 31.174 | 0.609 | 1.909 | 0.806 | 0.198 | 2.710 | 1.818 | 3.500 | 1.391 | 2.526 | 1.250 | 0.075 | 0.250 | 0.096 | 0.667 | 0.074 | 0.115 | 0.333 | 1.125 | 0.346 | 1.500 | 0.273 | 0.214 | 0.375 | 0.600 | 1.000 | 0.500 | 1.000 |
| 102.481 | 30.921 | 0.606 | 1.591 | 0.645 | 0.173 | 2.323 | 1.364 | 3.000 | 1.043 | 2.211 | 1.000 | 0.066 | 0.188 | 0.077 | 0.400 | 0.056 | 0.077 | 0.200 | 0.750 | 0.173 | 0.643 | 0.136 | 0.171 | 0.300 | 0.300 | 0.600 | 0.333 | 0.700 |
| 103.736 | 32.316 | 0.586 | 1.527 | 0.700 | 0.121 | 2.183 | 1.427 | 2.950 | 1.043 | 1.895 | 1.000 | 0.050 | 0.119 | 0.048 | 0.213 | 0.028 | 0.069 | 0.160 | 0.413 | 0.127 | 0.236 | 0.164 | 0.116 | 0.143 | 0.234 | 0.420 | 0.217 | 0.490 |
| 103.886 | 32.497 | 0.585 | 1.273 | 0.484 | 0.148 | 1.935 | 1.136 | 2.000 | 0.696 | 1.579 | 1.000 | 0.057 | 0.125 | 0.058 | 0.267 | 0.037 | 0.077 | 0.133 | 0.375 | 0.115 | 0.429 | 0.136 | 0.129 | 0.225 | 0.240 | 0.500 | 0.267 | 0.600 |
| 101.621 | 29.387 | 0.567 | 0.955 | 0.403 | 0.103 | 1.579 | 0.918 | 2.150 | 0.696 | 1.579 | 0.750 | 0.040 | 0.056 | 0.035 | 0.333 | 0.017 | 0.058 | 0.087 | 0.375 | 0.029 | 0.171 | 0.136 | 0.090 | 0.210 | 0.156 | 0.350 | 0.200 | 0.470 |
| 101.703 | 30.176 | 0.545 | 1.145 | 0.355 | 0.123 | 1.355 | 1.023 | 1.500 | 0.696 | 0.947 | 0.750 | 0.047 | 0.063 | 0.038 | 0.333 | 0.019 | 0.077 | 0.067 | 0.375 | 0.058 | 0.214 | 0.136 | 0.086 | 0.150 | 0.180 | 0.400 | 0.200 | 0.500 |
| 102.685 | 30.979 | 0.531 | 0.636 | 0.323 | 0.073 | 1.161 | 0.682 | 1.000 | 0.348 | 1.263 | 0.500 | 0.028 | 0.063 | 0.038 | 0.133 | 0.019 | 0.038 | 0.067 | 0.375 | 0.058 | 0.214 | 0.136 | 0.086 | 0.150 | 0.180 | 0.400 | 0.200 | 0.500 |

**TABLE S5** Seven types of models used for modeling the relationship between productivity and suitability.

| **Model code** | **Model type** |
| --- | --- |
| a | y=a*exp(b*x) |
| b | y=a*exp(b*x)+c |
| c | y = a*x + b |
| d | y=a*x^^^2+b*x+c |
| e | y=a*ln(x)+b |
| f | y=a*x^^^b |
| g | y=a*x^^^b+c |
